# Antagonism of Tetherin Restriction of HIV-1 Release by Vpu Involves Binding and Sequestration of the Restriction Factor in a Perinuclear Compartment

**DOI:** 10.1371/journal.ppat.1000856

**Published:** 2010-04-08

**Authors:** Mathieu Dubé, Bibhuti Bhusan Roy, Pierre Guiot-Guillain, Julie Binette, Johanne Mercier, Antoine Chiasson, Éric A. Cohen

**Affiliations:** 1 Laboratory of Human Retrovirology, Institut de recherches cliniques de Montréal (IRCM), Montreal, Quebec, Canada; 2 Department of Microbiology and Immunology, Université de Montréal, Montreal, Quebec, Canada; University of Geneva, Switzerland

## Abstract

The Vpu accessory protein promotes HIV-1 release by counteracting Tetherin/BST-2, an interferon-regulated restriction factor, which retains virions at the cell-surface. Recent reports proposed β-TrCP-dependent proteasomal and/or endo-lysosomal degradation of Tetherin as potential mechanisms by which Vpu could down-regulate Tetherin cell-surface expression and antagonize this restriction. In all of these studies, Tetherin degradation did not, however, entirely account for Vpu anti-Tetherin activity. Here, we show that Vpu can promote HIV-1 release without detectably affecting Tetherin steady-state levels or turnover, suggesting that Tetherin degradation may not be necessary and/or sufficient for Vpu anti-Tetherin activity. Even though Vpu did not enhance Tetherin internalization from the plasma membrane (PM), it did significantly slow-down the overall transport of the protein towards the cell-surface. Accordingly, Vpu expression caused a specific removal of cell-surface Tetherin and a re-localization of the residual pool of Tetherin in a perinuclear compartment that co-stained with the TGN marker TGN46 and Vpu itself. This re-localization of Tetherin was also observed with a Vpu mutant unable to recruit β-TrCP, suggesting that this activity is taking place independently from β-TrCP-mediated trafficking and/or degradation processes. We also show that Vpu co-immunoprecipitates with Tetherin and that this interaction involves the transmembrane domains of the two proteins. Importantly, this association was found to be critical for reducing cell-surface Tetherin expression, re-localizing the restriction factor in the TGN and promoting HIV-1 release. Overall, our results suggest that association of Vpu to Tetherin affects the outward trafficking and/or recycling of the restriction factor from the TGN and as a result promotes its sequestration away from the PM where productive HIV-1 assembly takes place. This mechanism of antagonism that results in TGN trapping is likely to be augmented by β-TrCP-dependent degradation, underlining the need for complementary and perhaps synergistic strategies to effectively counteract the powerful restrictive effects of human Tetherin.

## Author Summary

Restriction factors are cellular proteins that interfere with the multiplication and transmission of viruses and are therefore important components of natural immunity. Tetherin (also known as BST-2) is a recently identified restriction factor that traps viruses at the cell-surface, preventing their release and thus infection of other cells. Viruses have, however, developed means to counteract this restriction factor. Viral protein U (Vpu) is an accessory protein encoded by HIV-1, the causative agent of AIDS. Vpu antagonizes Tetherin and consequently promotes the release of HIV-1 particles. A series of recent reports proposed that Vpu would induce the degradation of this restriction factor in order to overcome its anti-viral activity. Here, we report that Vpu is able to enhance HIV-1 release in absence of Tetherin degradation. Instead, we found that Vpu interacts with Tetherin and interferes with the transport of the restriction factor towards the cell-surface. This would lead to re-localization of Tetherin in an intracellular organelle called the *trans*-Golgi network, resulting in insufficient levels of Tetherin at the cell-surface to trap progeny viruses. This mechanism of antagonism that results in TGN trapping could be augmented by the induction of degradation to effectively counteract the powerful restrictive effects of human Tetherin. Further characterization of this mechanism will improve our understanding of host antiviral defenses as well as provide new targets for the development of novel anti-HIV drugs.

## Introduction

Recent advances in retrovirology have revealed that mammalian cells do not always provide a hospitable environment for the replication of viruses that parasitize them. It is indeed becoming increasingly clear that mammalian cells express a variety of molecules and activities that interfere with specific steps of the replication cycle of retroviruses and other viruses [1]. Among these so-called restriction factors, the cellular protein CD317/BST-2/HM1.24, also designated as Tetherin in reference to its ability to tether HIV-1 virions to infected cells, was recently identified as a potent inhibitor of the release step of retroviruses [2],[3]. Tetherin is a heavily glycosylated type II integral membrane protein with an unusual topology in that it harbors two completely different types of membrane anchor at the N- and C-terminus; it is composed of a short N-terminal cytoplasmic tail linked to a transmembrane anchor (TM), an extracellular domain that include three cysteine residues important for dimerization, a predicted coiled-coil and a putative C-terminal glycophosphatidyl-inositol (GPI)-linked lipid anchor that is believed to ensure incorporation of Tetherin into cholesterol-rich lipid rafts [4],[5]. Tetherin inhibits the release of widely divergent enveloped viruses, including members of the lentivirus (primate immunodeficiency viruses), gammaretroviruses (murine leukemia virus), spumaretrovirus (foamy virus), arenavirus (Lassa virus), filovirus (Ebola and Marburg virus) families as well as Kaposi's sarcoma herpesvirus (KSHV) [2],[3],[6],[7],[8],[9],[10]. This broad-spectrum inhibition of enveloped virus particle release by Tetherin indicates that this restriction is unlikely to require specific interactions with viral proteins. In that regard, recent evidence indicates that Tetherin configuration rather than primary sequences is critical for antiviral activity since an entirely artificial Tetherin-like protein consisting solely of domains from three proteins that were analogous to Tetherin in terms of size and topology but lacking sequence homology with native Tetherin, inhibited particle release in a manner strikingly similar to Tetherin [11]. Tetherin-mediated restriction of virus particle release is believed to occur at sites of virus particle assembly at the plasma membrane since a strong co-localization between Tetherin and nascent particles generated from retroviral or filoviral structural proteins was observed at the cell-surface [7],[12]. In fact, recent findings using the artificial Tetherin-like protein support a model of restriction in which Tetherin directly cross-links virions to the plasma membrane [11]. Under basal conditions, Tetherin is expressed in B and T cells, plasmacytoid dendritic cells and myeloid cells and many transformed cell lines [2],[13],[14],[15],[16]. In addition, Tetherin expression is induced in many cell-types by type I and type II interferon (IFN), which suggests that it might be an important component of a broader antiviral innate immune defense [2],[13],[17]. In response to this restriction, many viruses express Tetherin antagonists such as KSHV K5, Ebola virus envelope glycoprotein (GP), simian immunodeficiency virus (SIVmac/smm) Nef, HIV-2 Env, SIVtan Env and HIV-1 viral protein U (Vpu), which was the first anti-Tetherin factor identified [2],[3],[9],[10],[18],[19],[20],[21],[22].

Vpu is an oligomeric type 1 integral membrane protein with two major activities during HIV-1 infection [23]. It contributes to the down-regulation of the CD4 receptor by targeting newly synthesized CD4 molecules that are bound to envelope glycoproteins (Env) in the endoplasmic reticulum (ER) for degradation by the ubiquitin-proteasome system [24],[25]. This degradation process relies on Vpu ability to associate with CD4 and to recruit β-TrCP, a component of the SCF^β-TrCP^ E3 ubiquitin (Ub) ligase, via phosphorylation of serines 52 and 56 within its DSGΦXS β-TrCP recognition motif [26],[27]. In addition, Vpu promotes HIV-1 particle release by suppressing human Tetherin activity in restrictive Tetherin-expressing cells such as epithelial cell lines (HeLa), T cell lines (Jurkat, CEM) and primary T lymphocytes and macrophages [2],[3],[17]. In contrast, no effect of Vpu is observed in permissive human cell lines devoid of Tetherin expression such as HEK 293T and HT1080. Interestingly, Vpu does not exert its anti-Tetherin activity in non-human cell lines regardless of their Tetherin expression levels [6],[28]. Indeed, although Tetherin variants found in rhesus macaques, African green monkeys (agm) and mouse cells are able to inhibit HIV-1 particle release, they are resistant to antagonism by HIV-1 Vpu [29],[30]. Analysis of Tetherin variants encoded by different species highlighted positively selected determinants in the Tetherin TM domain responsible for conferring sensitivity to Vpu antagonism [29],[30],[31],[32]. The mechanism by which Vpu counteracts Tetherin antiviral activity on HIV-1 particle release is still a matter of debate. Vpu was found to decrease the expression of Tetherin at the cell-surface [3] and to prevent Tetherin and Gag co-localization at sites of particle assembly [7],[12], suggesting that removal of Tetherin from its site of tethering action could underlie the mechanism by which Vpu counteracts this cellular restriction, although this model has lately been challenged [33]. Recently, a series of reports proposed proteasomal and/or endo-lysosomal degradation of Tetherin through a β-TrCP-dependent process as potential mechanisms by which Vpu antagonizes Tetherin antiviral activity [12],[20],[30],[31],[34],[35]. However, in all of these studies, Vpu-induced Tetherin degradation did not entirely account for the anti-Tetherin activity of Vpu. Thus, the precise mechanism(s) through which Vpu antagonizes Tetherin is yet to be elucidated.

In this study, we investigated the effect of Vpu on Tetherin expression and trafficking to obtain a better insight into the mechanism through which Vpu antagonizes Tetherin-mediated restriction of HIV-1 particle release. Here, we provide evidence that Vpu can promote HIV-1 particle release without affecting the total steady-state levels or the turnover rate of Tetherin. We further show that even though Vpu did not enhance Tetherin internalization from the plasma membrane, it did significantly slow-down the transport of the restriction factor towards the cell-surface. Notably, expression of Vpu led to a specific removal of cell-surface Tetherin and a re-localization of the residual pool of Tetherin to a perinuclear compartment that extensively overlapped with the TGN. Finally, we show that Vpu and Tetherin associate most probably via their TM domains and provide evidence that this association is necessary to relocate Tetherin from the cell-surface to the TGN and to counteract its restrictive activity on HIV-1 release. Overall, our results are consistent with a model whereby antagonism of Tetherin by Vpu involves sequestration of the restriction factor in a perinuclear compartment, away from virus assembly sites on the plasma membrane, a process that could be augmented by the induction of Tetherin degradation.

## Results

### Vpu can promote HIV-1 particle release without altering the steady-state levels or turnover of Tetherin

**Figure 1:**
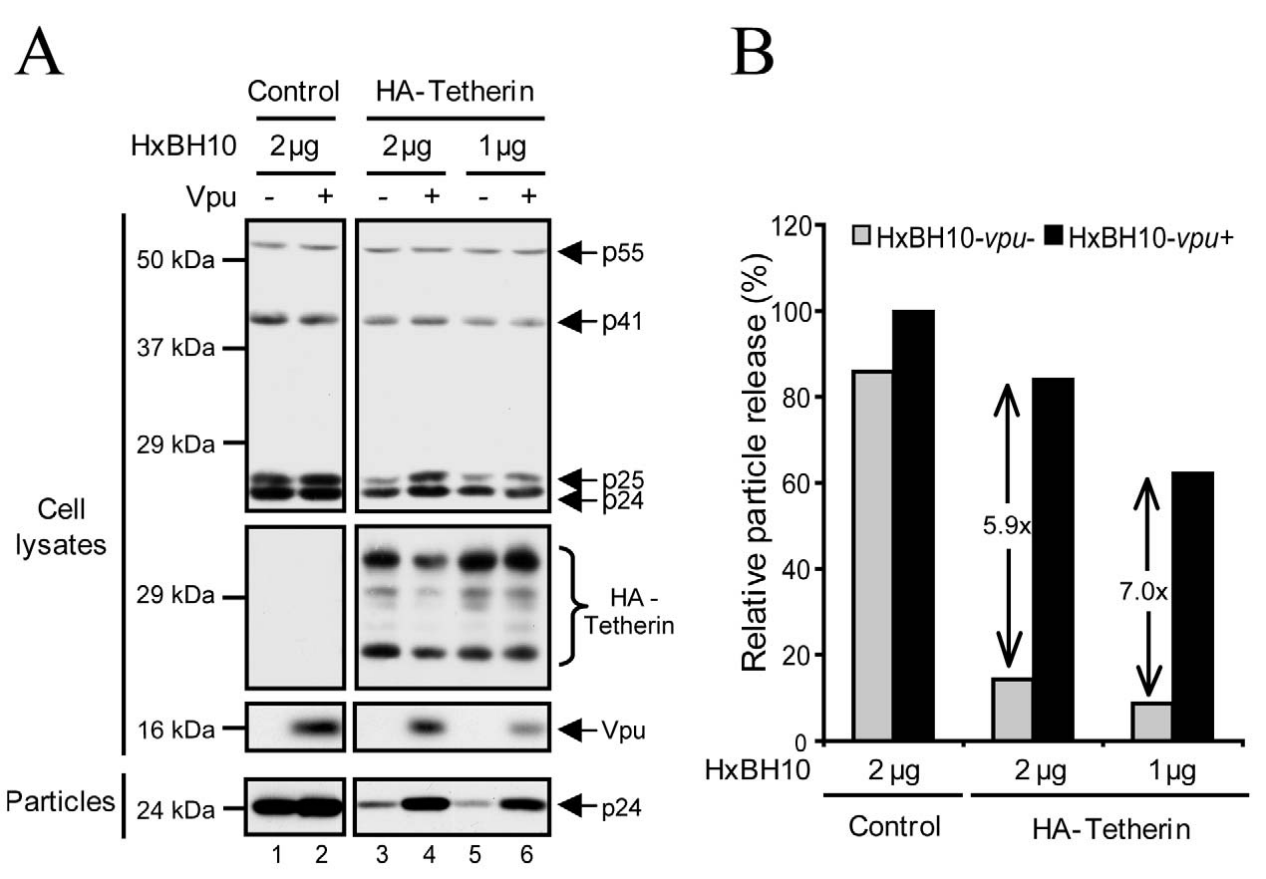
Enhancement of viral particle release in absence of Vpu-dependent reduction of Tetherin steady-state levels. HEK 293T cells were co-transfected with a fixed amount of the HA-Tetherin-expressing plasmid (235 ng) and the indicated amounts of HxBH10 proviral constructs. Forty-eight hours post-transfection, cells and virus-containing supernatants were harvested and processed for western blot analysis. (A) Proteins in the viral and cell lysates were detected using specific Abs. (B) Quantification of HIV-1 release efficiency. Bands corresponding to Gag products in cells and viral particles in panel (A), as detected using anti-p24 Abs, were scanned by laser densitometry. The virus particle release efficiency was determined as described in the Materials and Methods and calculated as a percentage of the release of HxBH10-*vpu+* (100%) in absence of HA-Tetherin. The values within the graph represent the fold increase of virus particle release upon Vpu expression.

To assess whether the reduction of Tetherin levels by Vpu was necessary and sufficient to promote efficient HIV-1 particle release, we analyzed the steady-state levels of exogenously-expressed HA-Tetherin in permissive HEK 293T cells in conditions where varying levels of virally-encoded Vpu was co-expressed (Fig. 1A). This cellular system was previously used to evaluate the effect of Vpu on Tetherin steady-state levels [30],[31],[34]. At low Vpu expression levels (1 µg of Vpu+ proviral construct), the levels of Tetherin were essentially similar to those detected in absence of Vpu (1 µg of Vpu-defective proviral construct) (compare lane 5 with lane 6), while at higher levels of Vpu expression (2 µg of Vpu+ proviral construct), they were significantly reduced (compare lane 3 with lane 4). As expected, ectopic expression of HA-Tetherin strongly inhibited the release of Vpu-defective HIV-1 particle relative to the Tetherin-negative control as demonstrated by the drastic reduction of virion-associated p24 in the supernatant (Fig. 1A, compare lanes 3 and 5 with lane 1; quantified in Fig. 1B). Interestingly, although Vpu did not affect the total levels of exogenous HA-Tetherin at low concentration, it still promoted efficient release of HIV-1 particle (Fig. 1A, compare lane 5 with lane 6; quantified in Fig. 1B). These results suggest that Vpu can reduce the total levels of Tetherin, yet this process does not appear to be absolutely necessary to promote HIV-1 particle release.

**Figure 2:**
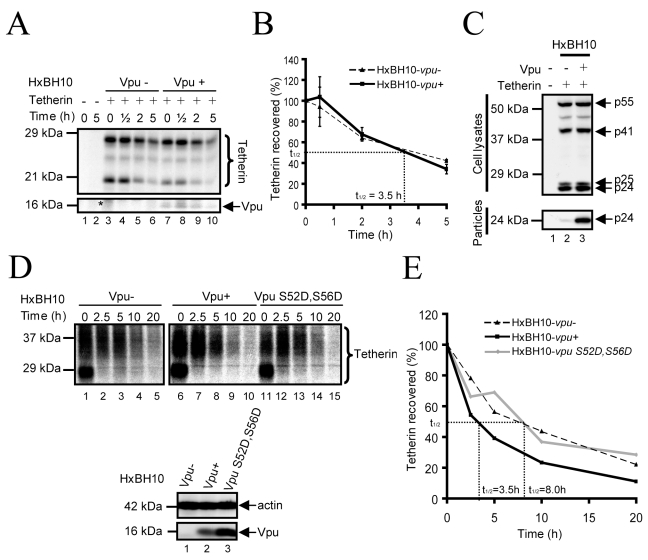
Analysis of the turnover of endogenous and exogenously-expressed Tetherin in the presence of Vpu. (A-C) Turnover of exogenously-expressed Tetherin. (A) HEK 293T cells were co-transfected with the indicated HxBH10 proviral constructs and the pcDNA-Tetherin plasmid. Forty-eight hours post-transfection, cells were pulse-labeled and chased for the indicated time intervals. Tetherin and Vpu were immunoprecipitated with specific Abs, and analyzed by autoradiography. The asterisk indicates the presence of a non-specific signal. (B) Quantitation of (A). The graph represents the percentage of Tetherin recovered relative to time 0. The signal intensity of all Tetherin-specific bands was determined for each time point by densitometric scanning. The percentage of Tetherin recovered was calculated as the ratio of the Tetherin bands signal at a given time relative to the signal at time 0. Error bars indicate the standard deviation of the mean from 2 independent experiments. (C) Virus-containing supernatants and a fraction of the cells from (A) were collected prior to labeling. Gag proteins from the cell and viral lysates were analyzed by western blot using anti-p24 Abs. (D-E) Turnover of endogenous Tetherin. (D) HeLa cells were infected with the indicated VSV-G-pseudotyped HxBH10 virus. Forty-eight hours post-infection, cells were pulse-labeled and chased for the indicated time intervals. Tetherin was immunoprecipitated with specific Abs, and analyzed by autoradiography. Vpu and actin were detected by western blot from cell lysates harvested prior to labeling. (E) Quantitation of (D) as described in (B). Full black line: HxBH10-*vpu+;* Dashed line: HxBH10-*vpu-;* Full grey line: HxBH10-*vpu S52D,S56D*.

To further confirm these observations, we analyzed the turnover of exogenously-expressed native Tetherin in condition of efficient Vpu-mediated virus particle release by pulse-chase labeling analysis (Fig. 2A-C). HEK 293T cells were co-transfected with the proviral constructs HxBH10-*vpu-* or HxBH10-*vpu+* and with a plasmid encoding native Tetherin. Forty-eight hours post-transfection, cells were pulse-labeled, chased for different intervals of time and analyzed for Tetherin and Vpu expression levels by sequential immunoprecipitation using specific antibodies (Abs). In parallel, transfected cells as well as virus-containing supernatants were collected prior to radio-labeling to monitor HIV-1 particle release by western blot. Tetherin-specific bands ranging from ∼20 kDa to ∼29 kDa and likely representing putative glycosylated forms of monomeric Tetherin were immunoprecipitated (Fig. 2A). Ectopic Tetherin turnover was not altered by Vpu since none of the Tetherin-specific bands showed any significant accelerated reduction over time in the presence of the viral protein (Fig. 2A; compare lanes 7–10 with lanes 3–6). Quantitative analysis of Tetherin turnover revealed that exogenous Tetherin has a half-life of approximately 3.5 h regardless of the presence of Vpu (Fig. 2B). Importantly, this lack of effect of Vpu on Tetherin turnover was observed in conditions of efficient Vpu-mediated HIV-1 particle release (Fig. 2C).

We further evaluated the half-life of endogenous Tetherin in infected HeLa cells in the presence or absence of Vpu. Vesicular stomatitis virus glycoprotein G (VSV-G)-pseudotyped HxBH10-*vpu*- or HxBH10-*vpu+* virus-infected HeLa cells were pulse-labeled, chased for different intervals of time and analyzed for Tetherin expression levels as described above. In this system, endogenous mature Tetherin was detected as a ∼30–37 kDa smear (Fig. 2D). A lower ∼27 kDa band, distinct from the predicted *M*r of 20 kDa for unglycosylated Tetherin, was also detected at time 0 and most probably corresponds to immature glycosylated forms of newly synthesized Tetherin still residing in the ER. Exogenously- and endogenously-expressed Tetherin were recently reported to display distinct mobilities (∼20–29 kDa (Fig. 2A) vs ∼27 and 30–37 kDa (Fig. 2D)) because they undergo different types of carbohydrate modifications [36]. Indeed, as demonstrated by Andrew and colleagues, we found that treatment of exogenous and endogenous Tetherin with Peptide: N-Glycosidase F (PNGase), an enzyme that cleaves all N-linked oligosaccharides, resulted in both cases in deglycosylated Tetherin proteins with a *M*r of 19–20 kDa that were recognized by our anti-Tetherin serum (data not shown).

Figure 2D reveals that mature endogenous Tetherin has a half-life (t_½_) of approximatively 8h (Fig. 2D, lanes 1–5; quantified in Fig. 2E), which is indeed longer than exogenously-expressed Tetherin (t_½_: 3.5 h). Tetherin turnover was accelerated in presence of Vpu (t_½_: 3.5 h) (compare lanes 6–10 to lanes 1–5; quantified in Fig. 2E), consistent with recent results reported by Douglas and colleagues using HeLa cells transduced with Vpu-expressing adenoviral vectors [20]. Since Vpu-mediated Tetherin degradation was reported to rely on its capacity to recruit β-TrCP [12],[20],[34],[35], we evaluated the turnover of the restriction factor in presence of the β-TrCP-binding defective Vpu S52D,S56D mutant. This mutant harbors mutations at the key amino-acids required for interaction with β-TrCP (Ser52, and Ser56 for Asp) and displays a phenotype very similar to the well-characterized Vpu S52N,S56N mutant [3],[12],[20],[27],[33],[35],[37],[38]. Notably, Vpu S52D,S56D is unable to mediate CD4 degradation [25] and to recruit β-TrCP (Fig. S1A), but is however able to partially down-regulate Tetherin from the cell-surface (Fig. S1B) and to promote HIV-1 particle release, albeit to a different extent than WT Vpu (Fig. S1C-D). Interestingly, even though Vpu S52D,S56D was still capable of promoting HIV-1 particle release, its expression did not affect Tetherin turnover (Fig. 2D, compare lanes 11–15 and lanes 1–5; quantified in Fig. 2E), consistent with the reported role of β-TrCP in Vpu-mediated Tetherin degradation [12],[20],[34],[35]. Taken together, these results provide evidence that Vpu can promote HIV-1 particle release without a detectable reduction of Tetherin intracellular levels or a notable modification of its turnover, suggesting that reduction of total levels of Tetherin by a degradative process may not be necessary and/or sufficient to fully explain the anti-Tetherin activity of Vpu.

### Vpu does not promote Tetherin endocytosis

**Figure 3:**
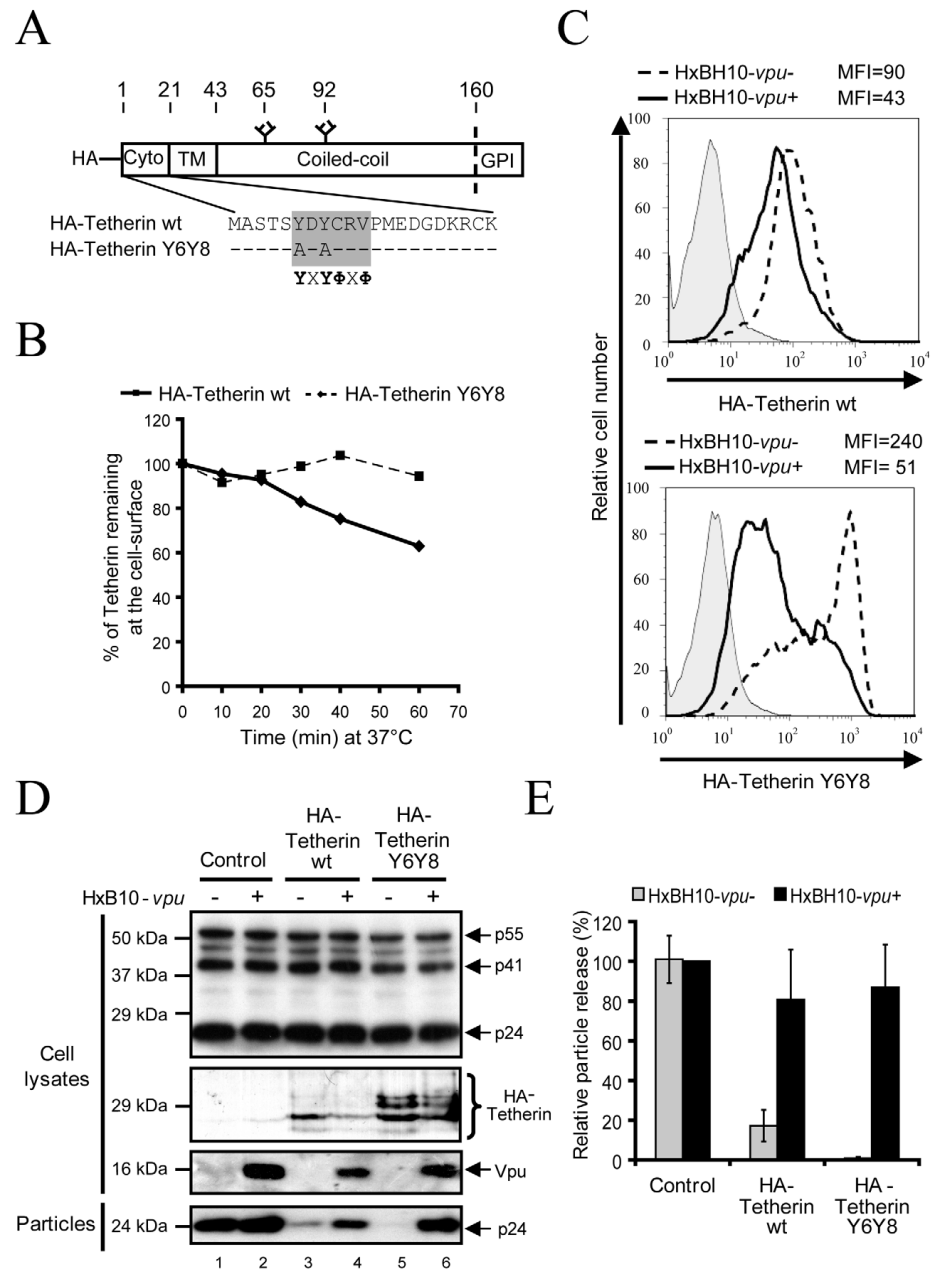
Mutation of Tetherin dual Tyrosine-based sorting motif does not affect sensitivity to Vpu. (A) Schematic representation of HA-Tetherin wt and HA-Tetherin Y6Y8. The overlapping Tyr-based motifs are presented in the grey box. X and Φ correspond to variable and hydrophobic amino-acid residues, respectively. Hyphens represent unchanged amino-acids. The site that is cleaved prior to addition of the GPI lipid anchor is represented by the dashed line. Glycosylation sites are represented at position 65 and 92. (B) Kinetics of HA-Tetherin Y6Y8 internalization. HEK 293T cells were co-transfected with the indicated HxBH10 constructs and a GFP-expressing plasmid. Forty-eight hours post-transfection, cell-surface Tetherin was labeled with Tetherin specific Abs at 4°C before incubating cells at 37°C for the indicated time intervals to allow endocytosis. Cells were then incubated at 4°C in presence of appropriate secondary Abs. The graph depicts the relative levels of Tetherin at the surface of GFP-expressing cells (time 0 = 100%) and represents the loss of Tetherin-specific signal following endocytosis. (C) Cell-surface expression of HA-Tetherin Y6Y8. HEK 293T cells were co-transfected with the indicated HA-Tetherin plasmid and HxBH10 proviral constructs in presence of a GFP-expressing plasmid. Cell-surface Tetherin expression was analyzed on GFP-positive cells by flow cytometry 48 h post-transfection. Geo mean values (depicted as MFI) are shown. Full lines: HxBH10-*vpu+*; dashed lines: HxBH10-*vpu-*; filled histogram: pre-immune control. The results are representative of two independent experiments. (D) Effect of HA-Tetherin Y6Y8 on HIV-1 particle release. HEK 293T cells were co-transfected with the indicated HxBH10 proviral constructs and HA-Tetherin-expressing plasmids. Forty-eight hours post-transfection, transfected cells and virus-containing supernatants were harvested. Proteins from cell and viral lysates were analyzed by western blot using specific Abs. (E) Quantitation of HIV-1 particle release. Bands corresponding to Gag products in cells and viral particles were scanned by laser densitometry. The virus particle release efficiency was determined as described in the Materials and Methods and calculated as a percentage of the release of HxBH10-*vpu+* (100%) in absence of HA-Tetherin. Error bars indicate the standard deviation of the mean from two independent experiments.

Rodent Tetherin is internalized from the plasma membrane and delivered back to the TGN through a clathrin-dependent pathway that requires the sequential action of AP2 and AP1 adaptor complexes [5]. Importantly, internalization was found to be dependent upon a dual tyrosine (Tyr)-based motif (YXXΦ, where Y corresponds to Tyr, the Xs are residues that are highly variable, and Φ corresponds to residues with bulky side chains) in the N-terminal cytoplasmic tail (amino acids at position 6 and 8) of the protein (Fig. 3A). One alternative mechanism to explain how Vpu down-regulates Tetherin cell-surface expression and counteracts its antiviral activity is by enhancing the rate of Tetherin endocytosis. To evaluate whether the natural pathway of Tetherin endocytosis was necessary for the anti-Tetherin activity of Vpu, we generated a mutant of Tetherin that harbored alanine substitutions at the two key Tyr residues within the dual Tyr-based internalization motif of the protein (HA-Tetherin Y6Y8) (Fig. 3A). Consistent with the previously reported role of this Tyr-based motif in rodent Tetherin endocytosis, substitution mutation of Tyr6 and Tyr8 prevented HA-Tetherin from being efficiently internalized from the cell-surface (Fig. 3B). To assess whether mutation of the Tyr-based motif affected Tetherin sensitivity to Vpu, HEK 293T cells were co-transfected with HxBH10-*vpu*- or HxBH10-*vpu+* proviral constructs and plasmids encoding for HA-Tetherin or HA-Tetherin Y6Y8. Even though HA-Tetherin Y6Y8 was expressed at higher levels at the cell-surface (mean fluorescence intensity (MFI) = 240) as compared to HA-Tetherin wt (MFI = 90), both proteins were down-regulated from the cell-surface by Vpu and indeed appeared to reach similar cell-surface steady state levels (MFI of 51 and 41, respectively) (Fig. 3C). HIV-1 particle release was also monitored by western blot, 48h post-transfection. Consistent with its higher cell-surface expression levels, the restriction of virus particle release was more pronounced in presence of HA-Tetherin Y6Y8 than with HA-Tetherin wt (Fig. 3D; compare lanes 5 and 3 with lane 1; quantified in Fig. 3D). It is interesting to note that the mutant protein was overall expressed at higher levels than the WT protein (Fig. 3D, compare lanes 5 and 3). This is likely the result of the inefficient clearance of HA-Tetherin Y6Y8, which is not efficiently internalized from the cell-surface. Nevertheless, Vpu was still proficient at overcoming the restricting activity of HA-Tetherin Y6Y8 on HIV-1 particle release as demonstrated by the increased levels of virion-associated p24 released in the supernatant (Fig. 3D, compare lane 6 with lane 5; quantified in Fig. 3E). Similarly, a 2h treatment with 10 µM chlorpromazine, a drug that blocks clathrin-coated pit assembly at the plasma membrane [39], did not affect the ability of Vpu to overcome Tetherin-mediated restriction of HIV-1 particle release in HeLa cells (data not shown). Altogether, these results suggest that Vpu does not manipulate clathrin-mediated endocytosis, the natural pathway of Tetherin endocytosis, as a mean to deplete the restriction factor from the cell-surface or to antagonize its antiviral activity.

**Figure 4:**
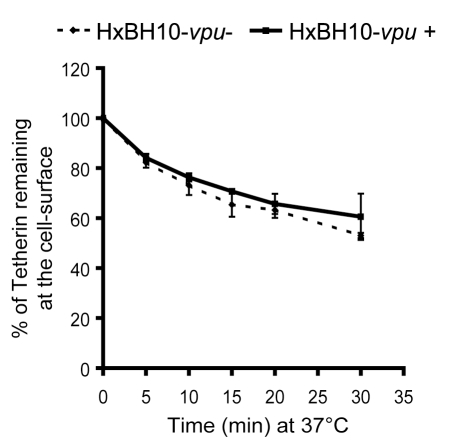
Vpu antagonizes Tetherin without promoting its endocytosis. HeLa cells were co-transfected with the indicated HxBH10 constructs and a GFP-expressing plasmid. Forty-eight hours post-transfection, cell-surface Tetherin was labeled with Tetherin specific Abs at 4°C before incubating cells at 37°C for the indicated time intervals to allow endocytosis. Cells were then incubated at 4°C in presence of the appropriate secondary Abs. The graph depicts the relative levels of Tetherin at the surface of GFP-expressing cells (time 0 = 100%) and represents the loss of Tetherin-specific signal resulting from endocytosis. Full lines: HxBH10-*vpu+*; dashed lines: HxBH10-*vpu-.* Error bars indicate the standard deviation of the mean from two independent experiments.

Since Vpu could accelerate Tetherin endocytosis by a clathrin-independent process, we next asked whether Vpu affected Tetherin internalization kinetics from the cell-surface using an assay that measures the contribution of all endocytosis pathways. To this end, we compared the rate of endocytosis of endogenous Tetherin in HeLa cells that were producing Vpu-positive HIV-1 (HxBH10-*vpu+*) with those producing a Vpu-defective virus (HxBH10-*vpu*-) (Fig. 4). Consistent with previous studies of rodent Tetherin [5], human Tetherin was internalized constitutively. Interestingly, even though Vpu down-regulated Tetherin expression on the cell-surface (data not shown), the internalization kinetics of cell-surface Tetherin was unaffected (Fig. 4). These results indicate that Vpu does not counteract Tetherin restriction by promoting Tetherin endocytosis.

### Vpu interferes with Tetherin trafficking to the cell-surface

**Figure 5:**
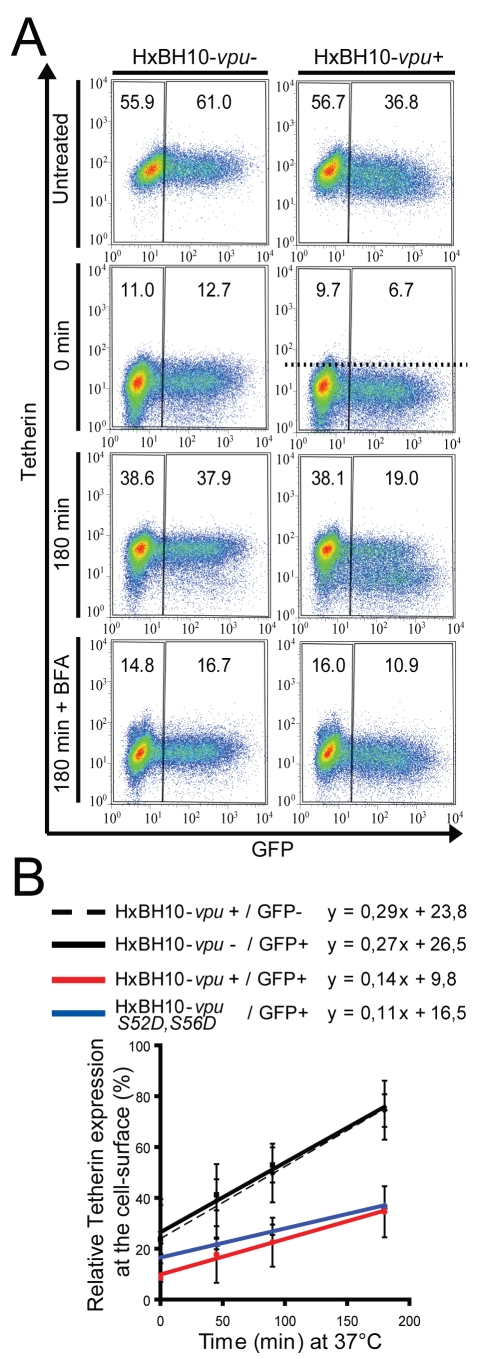
Vpu affects Tetherin trafficking to the cell surface. (A-B) HeLa cells were transfected with the indicated HxBH10 proviral constructs and a GFP-expressing plasmid. Forty-eight hours post-transfection, cells were harvested and treated with pronase (0.05%). After quenching of the proteolytic reaction, cells were cultured at 37°C for different time intervals and then immunostained for cell-surface Tetherin. (A) Dot plots representing cell-surface Tetherin levels relative to GFP expression. MFI values are presented for each gate. Treatment with BFA (10 µM) served as a positive control for intracellular retention. BFA was added to the media immediately after treatment with pronase and kept throughout the chase period. (B) Quantitative representation of panel (A). Kinetics of Tetherin re-expression were determined by calculating the percentage of Tetherin expression (MFI) at the surface of pronase-treated cells relative to the corresponding untreated GFP-negative control at different time intervals. Equations pertaining to each linear equation are shown above the graph. Error bars represent the standard deviation of at least two separate experiments. Black dashed line: HxBH10-*vpu+/*GFP*-*; black full line: HxBH10-*vpu-/*GFP*+*; red full line: HxBH10-*vpu+/*GFP*+*; blue full line: HxBH10-*vpu S52D,S56D/*GFP+. Similar results were obtained when the proportion of cells expressing Tetherin at the cell-surface was used as a read-out instead of Tetherin MFI. For these latter analyses, the cut-off was arbitrarily set on the pronase-treated HxBH10-*vpu+*/GFP+ time 0 sample (illustrated by the dotted line in Figure 5A) so that the background level of Tetherin-positive cells corresponded to 1%.

We have recently reported that regulation of HIV-1 release correlates with co-localization of Vpu and Tetherin in the TGN, thus raising the possibility that Vpu could act intracellularly by affecting Tetherin trafficking [40]. A cell-surface Tetherin re-expression assay was developed to determine whether Vpu affects Tetherin trafficking to the cell-surface. Conceptually, this assay implies the loss of Tetherin epitopes at the cell-surface and their subsequent recovery over time as demonstrated previously for analysis of the *Mtv-1* Superantigen protein trafficking [41]. HeLa cells were co-transfected with the HxBH10-*vpu-* or HxBH10-*vpu+* proviral constructs as well as with a GFP-encoding plasmid to allow gating of transfected cells. Forty-eight hours post-transfection, cells were harvested and treated with pronase (0.05%) to proteolytically remove cell-surface protein epitopes. After quenching the proteolytic reaction, stripped cells were incubated at 37°C for different time intervals to allow protein intracellular trafficking and re-expression at the cell-surface and then stained at 4°C with anti-Tetherin Abs. Expression of Tetherin in transfected (GFP-positive) or untransfected (GFP-negative) subpopulations was analyzed by flow cytometry (Fig. 5). As expected, dot plots revealed that Tetherin levels were down-regulated by Vpu in the untreated cells (compare the GFP-positive/HxBH10-*vpu+* subpopulation MFI (MFI = 36.8) with those of the GFP-negative/HxBH10-*vpu+* (MFI = 56.7) or GFP-positive/HxBH10-*vpu-* (MFI = 61.0) subpopulations) (Fig. 5A, untreated). Pronase treatment markedly reduced the levels of Tetherin at the cell-surface, indicating that Tetherin epitopes were efficiently removed from the cell-surface. Interestingly, the levels of Tetherin detected at time 0 in the GFP-positive/HxBH10-*vpu+* subpopulation was still lower (MFI = 6.7) than those detected in the GFP-negative/HxBH10-*vpu+* (MFI = 9.7) or GFP-positive/HxBH10-*vpu-* (MFI = 12.7) subpopulations (Fig. 5A, time 0 min). Similar levels of Tetherin re-expression was detected at the cell-surface of GFP-positive/HxBH10-*vpu-* cells and GFP-negative cells after 180 min of incubation as demonstrated by the comparable MFI detected in the two subpopulations. In contrast, Vpu caused a substantial reduction in Tetherin re-expression after 180 min, as shown by the lower MFI value in the GFP-positive/HxBH10-*vpu+* population (MFI = 19) compared to GFP-negative/HxBH10-*vpu+* cells (MFI = 38.1) (Fig. 5A; 180 min). Treatment of cells with 10 µM Brefeldin A (BFA), a fungal metabolite that blocks protein sorting from the ER to the Golgi, prevented efficient Tetherin re-expression at the cell-surface both in transfected (GFP-positive) and untransfected (GFP-negative) cells, demonstrating the specificity of the re-expression assay (Fig. 5A, BFA). It is interesting to note that the absolute difference (Δ) in MFI detected between the Vpu-expressing cells and control cells (GFP-positive or negative/HxBH10-*vpu-* and GFP-negative/HxBH10-*vpu+*) was amplified after 180 min (Δ = ∼19) as compared to time 0 (Δ = ∼3–6), suggesting an effect of Vpu on cell-surface Tetherin re-expression kinetics. Indeed, the kinetics of Tetherin re-expression at the cell-surface, as measured by evaluating Tetherin levels (MFI) at the surface of pronase-treated cells relative to the corresponding untreated GFP-negative control over 180 min, increased linearly in the GFP-positive/HxBH10-*vpu-* and was indistinguishable from that of the GFP-negative/HxBH10-*vpu+* control (slope of 0.27–0.29; Fig. 5B). After 180 min, approximately 75% of cell-surface Tetherin was recovered in both cases. In contrast, in presence of Vpu only ∼35% of Tetherin expression was recovered at the cell-surface. Indeed, the kinetics of Tetherin re-expression in these Vpu-expressing cells was much slower than the controls (slope of ∼0.14; Fig. 5B). Interestingly, the kinetics of Tetherin re-expression was similarly delayed in presence of the Vpu S52D,S56D mutant (slope of ∼0.11), indicating that the observed effect of Vpu on Tetherin re-expression at the cell-surface is not the consequence of Tetherin degradation. Similar analysis based on the proportion of Tetherin-positive cells as a read-out (cut-off was arbitrarily set on the pronase-treated HxBH10-*vpu+*/GFP+ time 0 sample) revealed analogous results (data not shown). Thus, our results suggest that Vpu expression interferes with Tetherin trafficking along the secretory and/or recycling pathways.

### Vpu expression causes a re-localization of the cellular pool of Tetherin in a perinuclear compartment

**Figure 6:**
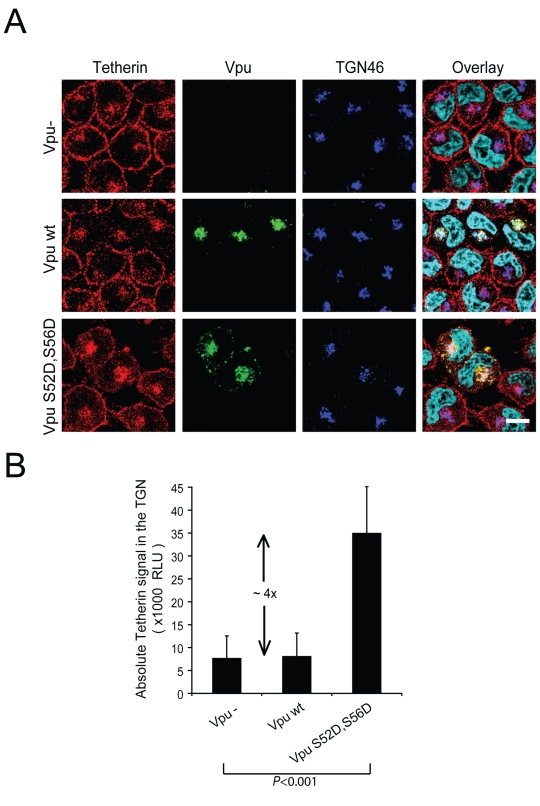
Vpu expression causes a re-localization of the cellular pool of Tetherin in a perinuclear compartment. (A) HeLa cells were infected with VSV-G pseudotyped HxBH10-*vpu-*, HxBH10-*vpu+* or HxBH10-*vpu S52D,S56D* virus. Forty-eight hours post-infection, cells were immunostained for cell-surface Tetherin, fixed, permeabilized and co-stained with anti-Tetherin (red), anti-Vpu (green) and anti-TGN46 (blue) specific Abs. Nuclei were counterstained with DAPI (cyan). Cells were observed by confocal microscopy. The white scale bar represents a distance of 10 µm. (B) Quantitation of the absolute Tetherin signal in the TGN was determined by measuring the specific Tetherin signal in the region delineated by the TGN46 marker on digital picture produced using similar acquisition time. Error bars indicate the standard deviation of the mean from the quantitative analysis of at least 25 distinct cells per conditions.

To further support these observations and identify the intracellular compartment where Tetherin might accumulate in presence of Vpu, we analyzed the intracellular distribution of endogenous Tetherin in VSV-G-pseudotyped HxBH10-*vpu*- and HxBH10-*vpu+* virus-infected HeLa cells by immunostaining and confocal microscopy. To this end, we developed a staining protocol, which allowed simultaneous detection of Tetherin at the cell-surface and in intracellular compartments as described in the Materials and Methods. In the absence of Vpu, Tetherin was detected primarily at the plasma membrane but also to a lower extent on internal membranes that overlapped partially with TGN46, a cellular marker of the TGN (Fig. 6, Vpu- panels), consistent with previous intracellular localization studies of rodent Tetherin [4],[13]. This localization pattern was drastically altered by the presence of Vpu, which caused an effective removal of Tetherin from the cell-surface without, however, significantly affecting the pool of proteins localized in the perinuclear compartment that co-stained with TGN46 (Fig. 6A, Vpu+ panels and Fig. 6B). Notably, as reported previously by our laboratory [40], Vpu and Tetherin co-localized extensively in the TGN. This altered localization pattern of Tetherin was not observed in neighbouring untransfected cells, which indeed displayed a strong Tetherin staining at the plasma membrane. Since we found that Vpu was accelerating Tetherin turnover in HeLa cells in a β-TrCP-dependent manner (Fig. 2D), we next analyzed the distribution of Tetherin in presence of the Vpu S52D,S56D mutant. In contrast to infected HeLa cells expressing WT Vpu, residual Tetherin was still readily detected at the plasma membrane of infected cells expressing the Vpu S52D,S56D mutant (Fig. 6A, Vpu S52D,S56D panels), a finding that most probably reflects the fact that this mutant is less efficient at down-regulating Tetherin from the cell-surface than WT Vpu (Fig. S1B). Interestingly, expression of this mutant resulted in a re-localization of the cellular pool of Tetherin in the TGN (Fig. 6A). Importantly, Vpu S52D,S56D caused an ∼4-fold increase of the absolute staining signal of Tetherin in the TGN relative to WT Vpu or the Vpu- control (Fig. 6B), suggesting that in absence of degradation, Vpu traps Tetherin in the TGN. Taken together, these microscopy studies suggest that HIV-1 Vpu promotes the sequestration of endogenous Tetherin in the TGN, most probably before triggering β-TrCP-dependent Tetherin degradation, thus preventing Tetherin's trafficking to the plasma membrane. Since these localization studies were performed in HIV-1-infected cells, these results further indicate that Tetherin sequestration occurs at physiological levels of Vpu expression.

### Association of Vpu and Tetherin involves their transmembrane anchor domains

**Figure 7:**
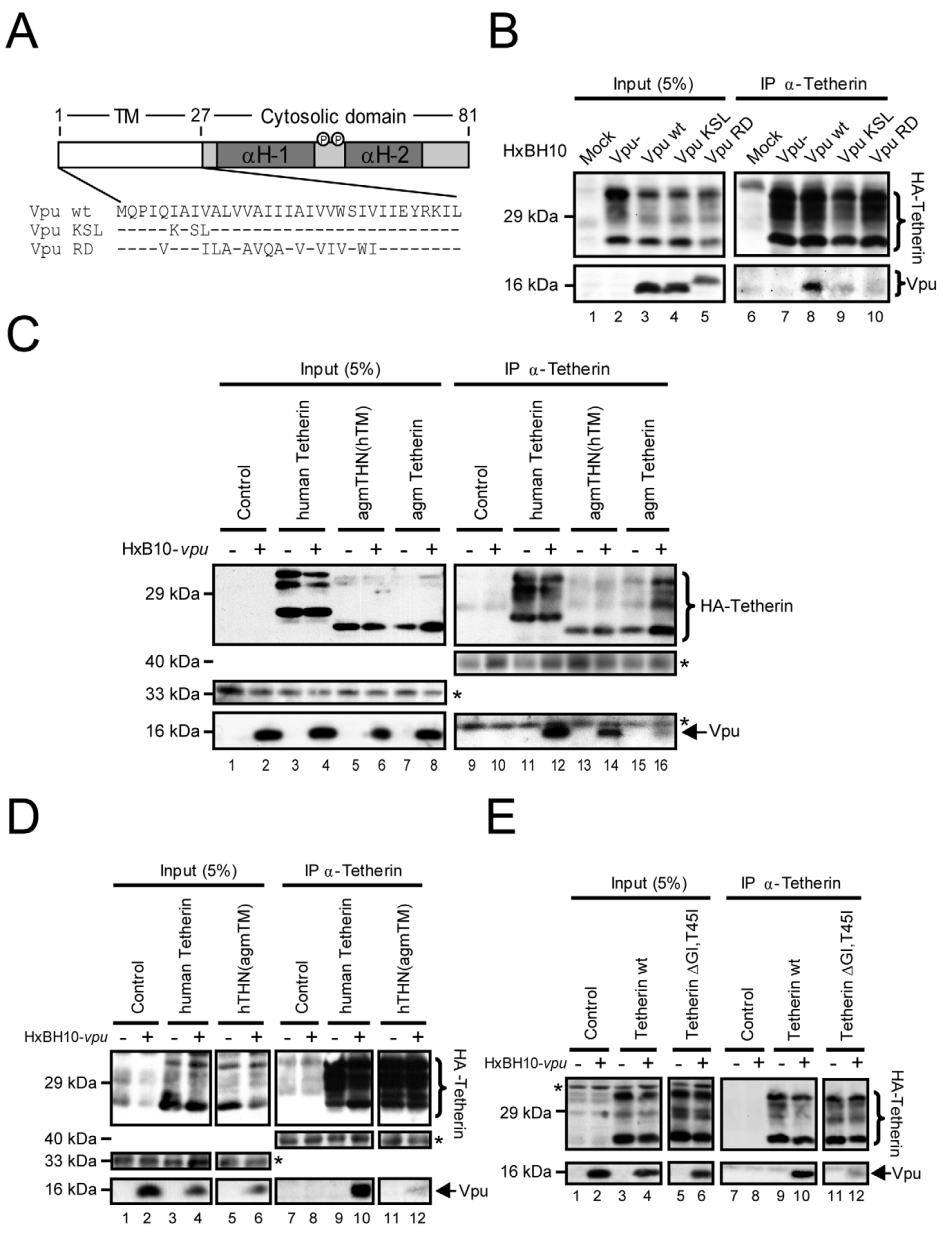
Association of Vpu and Tetherin involves their transmembrane anchor domains. (A) Schematic representation of WT Vpu, Vpu KSL and Vpu RD. The TM domain is represented by the unfilled box while the cytosolic domain with the two predicted α-helices (H-1 and H-2) is indicated by the filled box. Phosphorylation sites are represented by the small circles. The amino-acid sequence of the TM anchor region is represented below. Hyphens represent identical amino-acids. (B-E) Co-immunoprecipitation experiments of Tetherin and Vpu. (B) HEK 293T cells were transfected with a HA-human Tetherin-expressing plasmid and the indicated HxBH10-derived proviral constructs. Forty-eight hours post-transfection, cells were lysed and proteins were co-immunoprecipitated using anti-Tetherin Abs. Co-immunoprecipitated proteins were analyzed by western blot using anti-Tetherin and anti-Vpu Abs. (C-E) HEK 293T cells were transfected with the indicated HA-Tetherin-expressing plasmids and the specified HxBH10 proviral constructs. Forty-eight hours post-transfection, cells were lysed and proteins were co-immunoprecipitated and analyzed as described above. In (D), HA-Tetherin proteins were revealed by western blot using anti-HA Abs. Amounts of protein in the lysates prior to immunoprecipitation (input) are shown in the left panels. Non-specific bands, depicted by the asterisks, were used as loading controls.

Having shown that Vpu expression affects the intracellular trafficking of Tetherin to the cell-surface and promotes a sequestration of Tetherin in the TGN, we next assessed whether Vpu can associate with Tetherin. HEK 293T cells were co-transfected with the HxBH10-*vpu-* or HxBH10-*vpu+* proviral constructs as well as with a plasmid encoding HA-tagged Tetherin. Forty-eight hours post-transfection, cells were lysed in stringent detergent conditions with RIPA-DOC lysis buffer to avoid unspecific association resulting from membrane bridging. Tetherin was then immunoprecipitated from cell lysates with anti-Tetherin antibodies and the immunocomplexes were analyzed for the presence of Vpu by western blot. Immunoprecipitation of HA-Tetherin led to a selective pull-down of the Vpu protein, suggesting that Vpu can directly or indirectly interact with Tetherin in cells where it can antagonize Tetherin antiviral activity (Fig. 7B, lane 8). Early studies aimed at mapping the regions of Vpu necessary for enhancing HIV-1 release identified the TM domain of the protein as an important functional determinant since Vpu mutants that contained a randomized TM region (Vpu RD) or harbored point mutations within the TM spanning domain, such as Vpu KSL, failed to enhance virus particle release, yet were still stable, properly localized in a perinuclear compartment and able to induce efficient CD4 degradation [42],[43]. Vpu KSL contains a three amino-acid substitution in which Ile6, Ile8, and Val9 were replaced by Lys, Ser, and Leu, respectively, and contains a positively charged amino-acid in an area that is devoid of charge. To evaluate whether Vpu interacted with Tetherin through the TM anchor domain, we performed similar co-immunoprecipitations in HEK293T cells co-transfected with a plasmid encoding HA-tagged Tetherin and proviral constructs encoding Vpu RD (HxBH10-*vpu RD*) or Vpu KSL (HxBH10-*vpu KSL)* (Fig. 7A). In contrast to WT Vpu, both the Vpu RD and Vpu KSL mutants failed to co-immunoprecipitate efficiently with HA-Tetherin (Fig. 7B, compare lanes 9 and 10 with lane 8), suggesting that the association between the two proteins involves the TM domain of Vpu.

Since the TM of Vpu represents an important determinant for the association with Tetherin, we next determined whether the TM domain of Tetherin was also involved. Tetherin TM was recently proposed to contain the determinants responsible for the species-specific sensitivity to Vpu [29],[31],[32]. Notably, reciprocal exchange of TM domains between human (h) and rhesus monkey Tetherin (THN) proteins conferred sensitivity and resistance to Vpu and alterations in the human Tetherin TM domain that correspond to differences found in rhesus and agm Tetherin proteins were sufficient to render human Tetherin completely resistant to HIV-1 Vpu [29],[31],[32]. We constructed a set of expression plasmids encoding HA-tagged Tetherin chimeras with reciprocal exchanges of the TM between the human and agm proteins (Fig. S2A). In addition, we generated an expression plasmid encoding a HA-tagged human Tetherin that harbors double mutations in the TM domain (HA-human Tetherin ΔGI,T45I) comprising a deletion of Gly25 and Ile26 residues and a substitution of Thr45 for an Ile (Fig. S2A). This mutant was previously reported to strongly inhibit HIV-1 particle release but was completely resistant to antagonism by Vpu [29]. HEK 293T cells were co-transfected with the HxBH10-*vpu-* or HxBH10-*vpu+* proviral constructs as well as with the indicated HA-Tetherin-encoding plasmids (Fig. 7C-E). Forty-eight hours post-transfection, Tetherin was immunoprecipitated from cell lysates and immunocomplexes were further analyzed for the presence of Vpu by western blot. Since HA-agm Tetherin and the HA-agmTHN(hTM) chimeras were less expressed and/or detected using our anti-human Tetherin Abs (these chimeras were not efficiently precipitated with anti-HA Abs), Vpu association was compared between Tetherin variants displaying similar expression profiles. As shown in Figure 7C, immunoprecipitation of HA-human Tetherin co-precipitated Vpu (Fig. 7C, lane 12). As expected, HA-agm Tetherin, which was reported to be resistant to Vpu antagonism [29],[32], did not show a strong and specific association with Vpu. In contrast, replacement of the agm Tetherin TM domain with that of human Tetherin (HA-agmTHN(hTM)) restored the association with Vpu (Fig. 7C, compare lane 14 with lanes 16 and 12). Exchange of the human Tetherin TM with that of agm Tetherin (HA-hTHN(agmTM)) led to a marked reduction of Vpu association despite comparable levels of Tetherin expression (Fig. 7D, compare lane 12 with lane 10). Finally, introduction of the ΔGI,T45I mutations in the TM domain of HA-human Tetherin drastically reduced the association with Vpu (Fig. 7E, compare lane 12 with lane 10). These results suggest that the integrity of the TM domain of human Tetherin is necessary for the association with Vpu. Taken together, our data suggests that association of Vpu and human Tetherin involves their TM anchor domains.

### Association of Vpu to Tetherin is required to down-regulate the expression of the restriction factor at the cell-surface and to promote HIV-1 particle release

**Figure 8:**
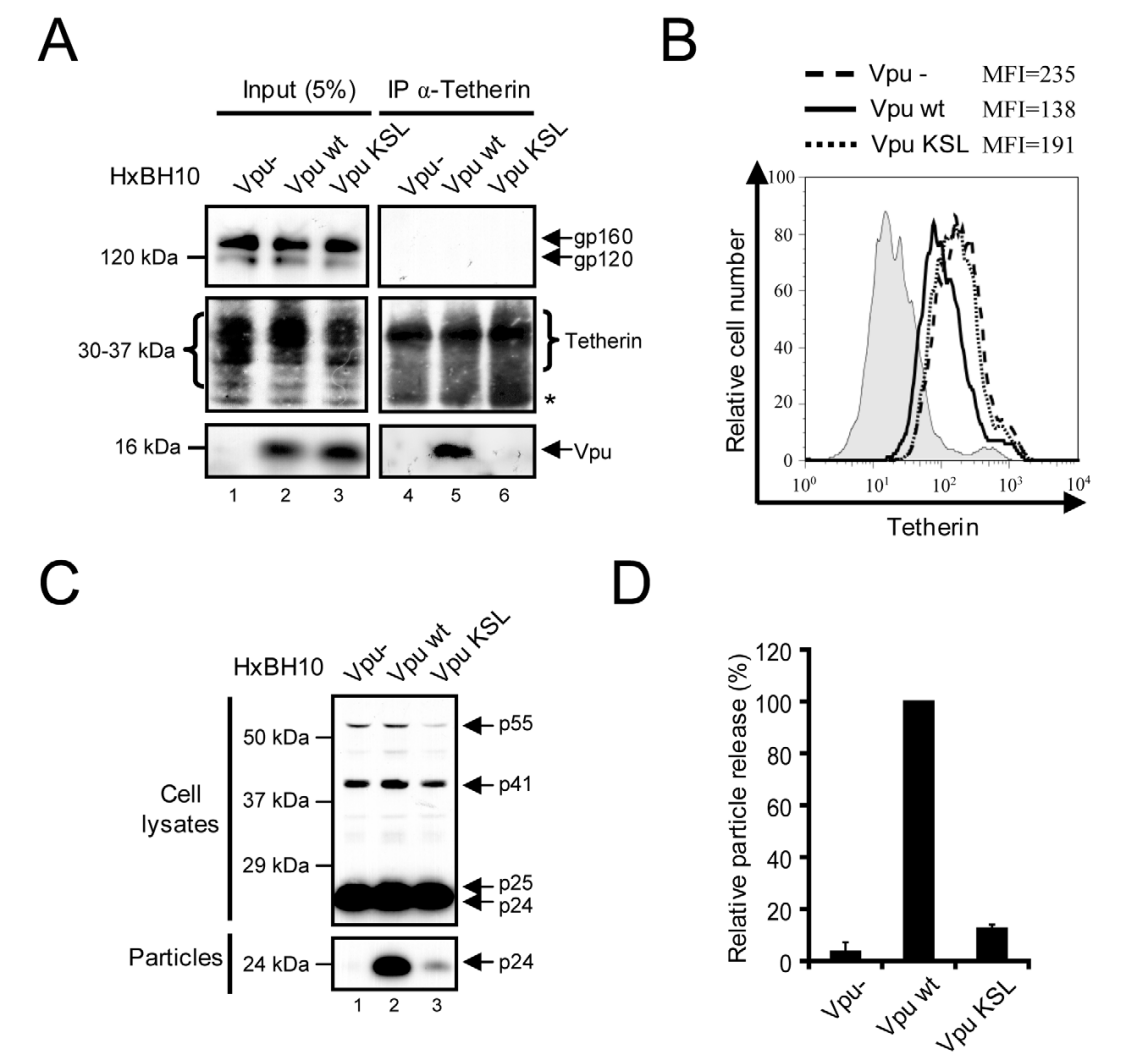
Association of Vpu to Tetherin is required to antagonize the antiviral activity of the restriction factor. (A) Association of Vpu with endogenous Tetherin. HeLa cells were transfected with the indicated HxBH10 proviral constructs. Forty-eight hours post-transfection, cells were lysed and proteins were co-immunoprecipitated using anti-Tetherin Abs. Co-immunoprecipitated proteins were analyzed by western blot using anti-Tetherin, anti-Vpu and anti-Env Abs. The amount of proteins in the lysate prior to immunoprecipitation (input) is shown in the left panel. Non-specific bands, depicted by the asterisks, were used as loading controls. (B) Cell-surface expression of Tetherin. HeLa cells were transfected with the indicated HxBH10 proviral constructs and a GFP-expressing plasmid. Cell-surface Tetherin expression was analyzed on GFP-positive cells by flow cytometry, 48h post-transfection. MFI values are shown beside the histogram. Filled histogram: pre-immune control; dashed line: HxBH10-*vpu*-; full line: HxBH10-*vpu*+; dotted line: HxBH10-*vpu KSL.* (C) Effect of Vpu on HIV-1 particle release. Cells and virus-containing supernatants were collected from the experiment described in (A), lysed and analyzed for the detection of Gag-related products by western blot using specific Abs. (D) Quantitation of virus particle release. Bands corresponding to Gag products in cells and virus particles were scanned by laser densitometry. The virus particle release efficiency was determined as described in the Materials and Methods and calculated as a percentage of the HxBH10-*vpu+* virus release (100%). The error bars represent the standard deviation from the mean of three independent experiments.

We next assessed whether association of Vpu to Tetherin was required to counteract Tetherin antiviral activity (Fig. 8). To do so, we transfected a proviral construct encoding WT Vpu or the Vpu KSL mutant in HeLa cells and first assessed their ability to associate with endogenous Tetherin. Wild type Vpu co-precipitated with endogenous Tetherin while Vpu KSL did not, confirming the results obtained when exogenous HA-tagged Tetherin was overexpressed in HEK293T cells expressing HxBH10-*vpu+* or HxBH10-*vpu KSL* proviruses (Fig. 8A, compare lane 6 with lane 5). Association of Vpu with endogenous Tetherin was specific since HIV-1 envelope glycoprotein precursor gp160, another type 1 integral protein, did not co-precipitate with Tetherin (Fig. 8A). Interestingly, the Vpu KSL mutant was strongly attenuated in its ability to down-regulate the steady-state levels of Tetherin at the cell-surface relative to WT Vpu since Tetherin cell-surface expression in presence of Vpu KSL was significantly higher than in presence of WT Vpu (MFI of 191 vs 138), yet still slightly lower than in the Vpu-negative control (MFI =  235) (Fig. 8B). Importantly, as previously reported [43], this mutant was drastically attenuated in its ability to promote efficient HIV-1 particle release (Fig. 8C, compare lanes 3 and 2 with lane 1; quantified in Fig. 8D). Similarly to Vpu KSL, the Vpu RD mutant, which is also defective for Tetherin binding, was also markedly attenuated in its ability to down-regulate Tetherin cell-surface expression and to promote efficient HIV-1 release in HEK 293T cells ectopically-expressing Tetherin (Fig. S3A and B), consistent with results reported by previous studies [3],[42]. Human Tetherin containing the agm TM domain (HA-hTHN(agmTM)) or the ΔGI,T45I mutations (HA-human Tetherin ΔGI,T45I) lost the ability to bind Vpu (Fig. 7D and E) and as expected still restricted HIV-1 particle release even in the presence of Vpu (Fig. S2B and C for HA-hTHN(agmTM) and Fig. S2D and E for HA-human Tetherin ΔGI,T45I). Unexpectedly, introduction of the human Tetherin TM domain in agm Tetherin (HA-agmTHN(hTM), did not reinstate a significant sensitivity to Vpu (Fig. S2 B and C), despite a detectable restoration of the Vpu binding (Fig. 7C). This functional phenotype (absence of Vpu sensitivity) is different from that obtained by McNatt and colleagues [29] using agm or rhesus Tetherins containing the human Tetherin TM domain but is similar to that reported by Goffinet and colleagues [30] using rodent Tetherin proteins containing the human Tetherin TM domain. Difference in the configuration of the TM domain in these Tetherin chimeric constructs may explain this discrepancy. The result obtained with the HA-agmTHN(hTM) chimeric construct suggests that association of Tetherin with Vpu is necessary but not sufficient to overcome Tetherin-mediated restriction of HIV-1 particle release. However, we cannot rule-out the possibility that the binding of Vpu to the HA-agmTHN(hTM) chimeric protein may indeed be less efficient than with HA-human Tetherin since our antibody may underestimate the levels of expressed HA-agmTHN(hTM) proteins (Fig. 7C, compare lane 14 and lane 12). Thus, alternatively, a threshold level of Vpu association to Tetherin may be necessary to antagonize the antiviral activity of the restriction factor. Nonetheless, overall, these results suggest that association of Vpu to Tetherin is required to antagonize the antiviral function of the restriction factor.

### Association of Vpu to Tetherin is critical for the re-localization of the restriction factor in the TGN

**Figure 9:**
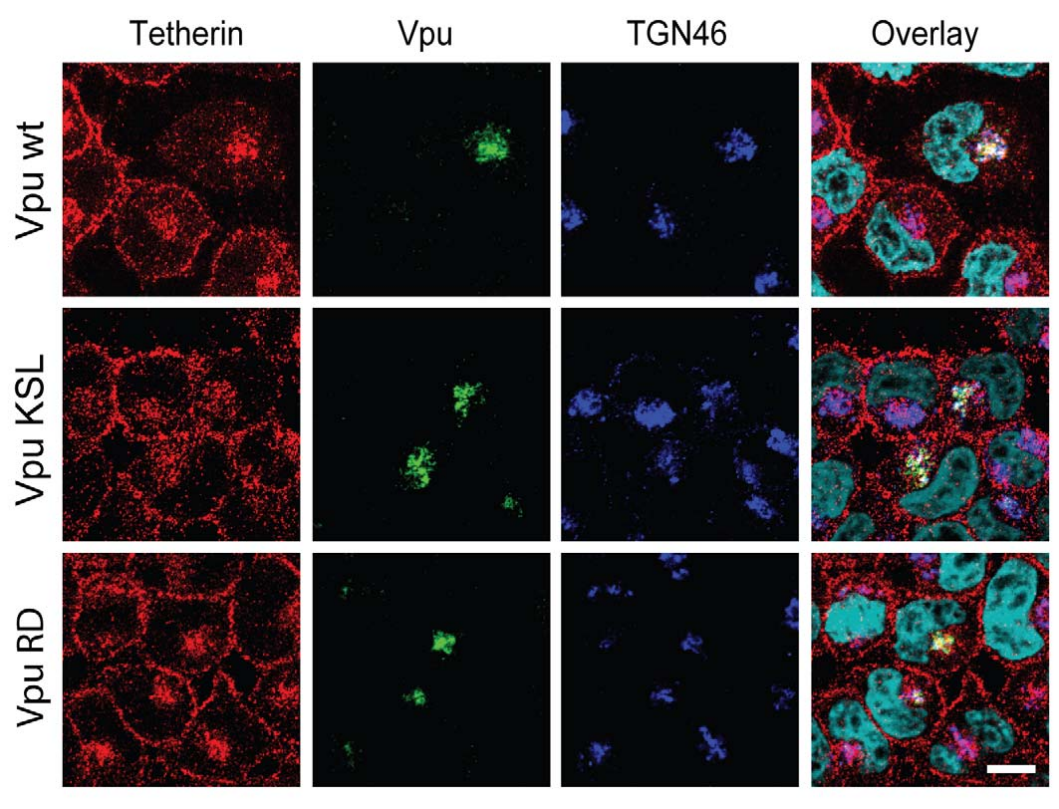
Re-localization of Tetherin in the TGN requires the association of Vpu to the restriction factor. HeLa cells were infected with the indicated VSV-G pseudotyped HxBH10-derived virus. Forty-eight hours post-infection, cells were immunostained for cell-surface Tetherin, fixed, permeabilized and co-stained with anti-Tetherin (red), anti-Vpu (green) and anti-TGN46 (blue) specific Abs. Nuclei were counterstained with DAPI (cyan). Cells were observed by confocal microscopy. The white bar represents a distance of 10 µm.

Since we showed that expression of Vpu could cause a re-localization of Tetherin from the plasma membrane to the TGN, we next tested whether the Vpu TM domain mutants, Vpu RD and Vpu KSL, which are unable to associate with Tetherin, could still sequester endogenous Tetherin in the TGN. Hela cells were infected with VSV-G-pseudotyped viruses expressing WT Vpu or Vpu RD or Vpu KSL and infected cells were immunostained and analyzed by confocal microscopy. Figure 9 reveals that both the Vpu RD and Vpu KSL lost the ability to efficiently remove Tetherin from the cell-surface and to relocate the restriction factor to the TGN. Indeed, quantitative analysis of the Tetherin signal localized in the TGN relative to the total cellular Tetherin signal showed that cells expressing the Tetherin degradation-defective Vpu S52D,S56D mutant displayed an increased proportion of Tetherin signal in the TGN compared to the Vpu- control. (∼57% relative to ∼18%; p<0.001) (Fig. 6). In contrast, cells expressing the Vpu RD or Vpu KSL mutants showed ∼25% of the Tetherin signal in the TGN, a proportion that was in fact just slightly higher than that found in cells infected with a Vpu-defective virus (Fig. 9). Accordingly, in contrast to Vpu S52D,S56D, these mutants did not show any increase in the absolute levels of Tetherin signal in the TGN (Fig. 9). These results indicate that Vpu promotes the sequestration of Tetherin in the TGN by a process that is dependent on the association of the two proteins. Taken together with the functional analysis and the co-precipitation experiments, these results further suggest that the antagonism of Tetherin function by Vpu involves binding and sequestration of the restriction factor in the TGN.

## Discussion

The Vpu accessory protein stimulates the release of HIV-1 virions by antagonizing a restriction on virus particle release mediated by Tetherin at the cell-surface [2],[3]. This antagonism appears to closely correlate with the ability of Vpu to mediate down-regulation of Tetherin expression from the cell-surface [3],[12],[20]. While this down-regulation is accompanied by enhanced degradation of Tetherin in several infected cell types, such as Jurkat [20] and CEM-G37 T cells [21] or macrophages [33], several lines of evidence also suggest that degradation of Tetherin *per se* cannot entirely account for Vpu-mediated counteraction of the restriction factor since: 1) Vpu decreased total cellular Tetherin to a lesser extent than cell-surface Tetherin in HeLa cells [12]; 2) Vpu expression did not result in a reduction of intracellular Tetherin in infected CEMx174 and H9 cells, yet virus replication in these cells was Vpu-responsive [33]; 3) Vpu mutants that contained substitution mutation in the DSGΦXS β-TrCP recognition motif that rendered them deficient for directing β-TrCP-dependent degradation of Tetherin were still able to partially [3],[12],[20],[34],[37] (Fig. S1C and D) or in some instances totally [33],[44] overcome the particle release restriction; 4) binding of Vpu to Tetherin was recently shown to be sufficient for a partial relief of the restriction [20],[34].

We show here that Vpu can promote efficient HIV-1 particle release without a detectable reduction of the total steady-state levels of Tetherin (Fig. 1) nor a notable modification of the restriction factor turnover rate in transfected HEK 293T cells (Fig. 2A -C), suggesting that degradation of the antiviral factor *per se* is not necessary and/or sufficient to account for Tetherin antagonism at least in this experimental system. Interestingly, Vpu expression in HeLa cells increased endogenous Tetherin turnover (Fig. 2D and E), as reported previously by Douglas and colleagues [20]. This Vpu-mediated degradation process was however still relatively slow (half-life of Tetherin decreases from ∼8 h to 3.5 h in presence of Vpu) as compared to the very efficient CD4 receptor degradation induced by Vpu (half-life of CD4 decreases from ∼6 h to ∼12 min in presence of Vpu) [45], and as such is unlikely to explain the powerful antagonism of Tetherin by Vpu. Thus, Vpu-mediated counteraction of Tetherin restriction must involve other mechanisms. Our results further indicate that Vpu does not promote endocytosis of Tetherin as a mechanism to antagonize the restriction factor. Indeed, mutation of the two critical tyrosine residues located within a dual tyrosine-based sorting motif in the cytoplasmic domain of the protein, did not abolish the sensitivity of Tetherin to Vpu, as was indeed recently reported by Iwabu and colleagues [35], yet led to an increased accumulation of the restriction factor at the cell-surface and to a more potent restriction of HIV-1 particle release (Fig. 3). Moreover, although analysis of Tetherin endocytosis kinetics showed that the protein is constitutively internalized, it did not reveal any increase in the rate of Tetherin endocytosis in presence of Vpu (Fig. 4), and as such confirmed the results recently reported by Mitchell and colleagues [12]. These results are consistent with previous findings showing that Vpu-mediated enhancement of virus particle release is not significantly affected by expression of dominant negative (DN) mutants of Dynamin or Rab5 nor by depletion of clathrin heavy chain or by treatment with inhibitors of endocytosis such as chlorpromazine (data not shown) [6],[40],[46]. Collectively, they indicate that Vpu does not antagonize Tetherin by enhancing its endocytosis from the cell-surface. Instead, these findings suggest that Vpu affects Tetherin anterograde trafficking events whose net effect is depletion of the restriction factor from the cell-surface. In support of this hypothesis, a previous report suggested that Vpu may acts as a regulator of protein transport along the secretory pathway [47]. In fact, upon analyzing the kinetics of Tetherin expression at the cell-surface of protease-treated HeLa cells, we noticed that Tetherin re-expression was significantly reduced in presence of Vpu (Fig. 5). It is important to note, here, that this effect is occurring regardless of whether Vpu is inducing Tetherin degradation in these experimental conditions since: 1) it is the rate of re-expression, as defined by the slope of the graph of Figure 5B that is affected in presence of Vpu; 2) re-expression of cell-surface Tetherin was also delayed in presence of the Tetherin degradation-defective Vpu S52D,S56D mutant (Fig. 5B). Consistent with this observation, examination of infected HeLa cells expressing Vpu revealed that the cellular pool of Tetherin was re-localized from the cell-surface to a perinuclear compartment that stained positive with the TGN marker TGN46 and Vpu itself (Fig. 6). Although some of the loss of cell-surface Tetherin in Vpu-expressing HeLa cells could be attributed to Vpu-mediated degradation of the restriction factor, similar experiments performed with the Vpu S52D,S56D mutant demonstrated that Vpu can cause a redistribution of Tetherin from the plasma membrane to the TGN. These results suggest that reduction of Tetherin levels at the plasma membrane involves a step whereby Vpu sequesters Tetherin in the TGN. They also provide a possible mechanism to explain the partial relief of Tetherin restriction observed by several groups upon expression of β-TrCP-binding defective Vpu mutants [12],[20],[34]. However, since residual Tetherin was still readily detected at the plasma membrane of HeLa cells expressing the Vpu S52D,S56D mutant (Fig. 6A and Fig. S1B), this sequestration may not be sufficient to effectively prevent Tetherin to reach the cell-surface at least in this model. These observations underline, perhaps, the need for β-TrCP-mediated trafficking and degradation processes as a complementary mechanism to efficiently remove Tetherin from the cell-surface, particularly in certain cell types or under specific conditions, such as IFN exposure, where cellular Tetherin expression levels are high. Thus, the different particle release phenotypes observed with Vpu mutants that are unable to recruit β-TrCP, such as Vpu S52D,S56D, (these range from as efficient than WT Vpu to a ∼50% attenuation) [3],[12],[20],[33],[34],[37],[44] may in fact relate to differing levels of Tetherin expression in the target cells. Although, it is conceivable that Vpu-mediated sequestration of Tetherin in the TGN may explain how Vpu antagonizes Tetherin in the absence of a decrease in total Tetherin expression, it still remains unclear how Vpu could antagonize Tetherin in the absence of down-regulation from the cell-surface and a decrease in total cellular expression in certain cell types [33].

We also confirmed by co-immunoprecipitation studies that Vpu interacts with Tetherin [20],[32],[34],[35] and demonstrated using well-characterized Vpu and Tetherin mutants as well as chimeric proteins between human and African green monkey Tetherin molecules that this physical interaction involves the transmembrane domains of the two proteins (Fig. 7 and Fig. 8). These findings are consistent with previous results showing that the transmembrane domain of Vpu is required for both the down-regulation of surface Tetherin and the enhancement of HIV-1 particle release [3],[42],[43]. They are also supported by recent data indicating that the inability of Vpu to antagonize the restrictive effect of African green monkey and rhesus Tetherin proteins is a consequence of amino-acid changes in the transmembrane domain of the rhesus and African green monkey protein relative to the human form [29],[32]. Importantly, our data establish a direct functional link between association of Vpu to Tetherin and Tetherin antagonism since we showed that Vpu's ability to interact with Tetherin was necessary: 1) to counteract the restriction on HIV-1 particle release and down-regulate Tetherin from the cell-surface (Fig. 8, Fig. S2 and S3); and, 2) to induce a re-localization of the cellular pool of Tetherin from the plasma membrane to the TGN (Fig. 9). Unexpectedly, our observation showing that introduction of the human Tetherin TM domain in African green monkey Tetherin did not reinstate a significant sensitivity to Vpu (Fig. S2B and C) despite a detectable restoration of the Vpu binding raises the possibility that this interaction may not be sufficient to explain Tetherin antagonism. The requirement for additional cellular factor(s) in the Vpu-mediated Tetherin antagonism is therefore a possibility that warrants further investigations.

**Figure 10:**
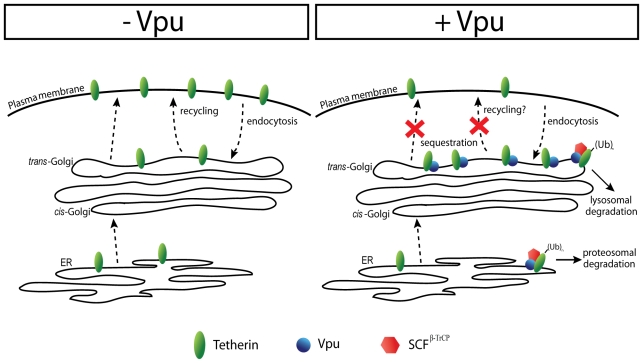
Model of Vpu-mediated down-regulation of Tetherin from the cell-surface. In absence of Vpu, Tetherin traffics along the secretory pathway and reaches the plasma membrane. The protein is endocytosed, transported to the TGN and is then recycled back to the cell-surface. In presence of Vpu, Tetherin is forming complexes with the viral protein in the membrane of the ER. Recruitment of SCF^β-TrCP^ by Vpu leads to ubiquitination and proteosomal degradation of a fraction of Tetherin. Tetherin escaping degradation exits the ER. In the membrane of the TGN, Vpu binds endocytosed Tetherin and/or Tetherin arriving from the ER and causes the sequestration of the restriction factor. Subsequently, Vpu induces Tetherin ubiquitination through the recruitment of SCF^β-TrCP^, thus targeting the ubiquitinated proteins to lysosomal degradation.

Since our co-immunoprecipitation and functional data indicate that a physical interaction between Vpu and Tetherin is required for Tetherin antagonism and sequestration, this raise the possibility that Vpu may simply interact with Tetherin and inhibit its outward trafficking from the TGN since all membrane proteins are transported to the plasma membrane through the TGN (Fig. 10). Since mutation of the dual Tyr signal in the Tetherin cytoplasmic tail does not abolish the sensitivity to Vpu, it appears that Tetherin sequestration in the TGN may occur before its endocytosis from the cell-surface and as such may involve newly synthesized Tetherin en route to the plasma membrane. However, we cannot completely rule-out that Vpu could interact with endocytosed Tetherin in the TGN and prevent its recycling back to the cell-surface, given that previous data from the Spearman group demonstrated a requirement for the recycling endosomes in Vpu function [48]. Furthermore, recent studies reported that AP-2 depletion [12] or over-expression of DN mutant of Dynamin (Dyn2-K44A) [35] could partially interfere with Vpu-mediated down-regulation of Tetherin expression from the cell-surface. Indeed, since Vpu is expressed from a Rev-dependent bicistronic mRNA encoding Env and consequently is made late during the virus life cycle [49], the direct removal of Tetherin from the plasma membrane via endosomal trafficking may be critical to ensure a rapid and efficient neutralization of the restriction on HIV-1 release. It is nevertheless surprising that mutation of the Tetherin dual Tyrosine-based endocytosis motif did not affect the ability of Vpu to counteract Tetherin-mediated restriction of HIV-1 particle release. Perhaps co-expressing transiently the two proteins simultaneously may not adequately reflect physiological conditions given that Tetherin is normally already present at the plasma membrane when Vpu is expressed. Alternatively, it is also possible that sequestration of newly synthesized Tetherin in the TGN may rapidly clear the restriction factor from its site of virion-tethering action at the plasma membrane depending on the physiologic rate of Tetherin turnover at the plasma membrane. More studies will be required to assess whether Vpu affects Tetherin trafficking at a pre- or/and post-endocytic step.

Vpu-mediated sequestration of Tetherin in the TGN could be complemented by β-TrCP-dependent degradation processes, thus enhancing Tetherin antagonism. It is conceivable that Vpu by forming a complex with β-TrCP and Tetherin in the membrane of the TGN could potentially induce ubiquitination via interaction with the SCF^β-TrCP^ E3 ubiquitin ligase. This ubiquitination event could either enhance the sequestration by preventing Tetherin recycling to the cell-surface or/and targets it to lysosomes for degradation (Fig. 10). Our data indicating that Vpu causes a re-localization of the cellular pool of Tetherin in the TGN, where indeed the two proteins strongly co-localize, suggests that Vpu affects Tetherin trafficking and potentially its degradation from a post-ER compartment(s), and as such is difficult to reconcile with a mechanism of proteasomal degradation of the antiviral factor through the cellular ER-associated degradation (ERAD) pathway [30],[31],[34]. However, given the fact that Vpu induces degradation of the CD4 receptor in the ER by an ERAD-like mechanism [25], it is still possible that some proteasomal degradation in the ER may contribute to Tetherin depletion. Whether Vpu and Tetherin trafficking cross each other in the TGN, thus permitting a physical interaction between the two proteins, remains to be determined. In this regard, it is interesting to note that recent data from our laboratory have shown that the ability of Vpu to suppress Tetherin-mediated restriction of HIV-1 particle release was linked to its localization in the TGN [40]. Further studies aimed at identifying the determinants regulating Vpu trafficking and localization in the TGN will likely shed light on this mechanism.

HIV-1 Vpu appears to share the ability to sequester Tetherin in the TGN with other Tetherin antagonists, namely HIV-2 Rod and SIVtan Env. Like HIV-1 Vpu, HIV-2 Rod Env was recently shown to interact with Tetherin and to cause a redistribution of Tetherin in the TGN, although in the case of HIV-2 Env, no evidence of Tetherin degradation was observed [21]. Similarly, SIVtan Env was also shown to downregulate cell-surface Tetherin by sequestering the restriction factor in intracellular compartment(s) [22]. Collectively, these findings suggest a common mechanism of antagonism that results in TGN trapping, which can be augmented by the induction of degradation in the case of Vpu. Given the powerful restrictive effects of human Tetherin on HIV production, this dual mechanism of antagonism mediated by Vpu may have provided HIV-1 with stronger countermeasures to antagonize Tetherin. This genetic and functional divergence between HIV-1 and HIV-2 may perhaps account for the different virulence properties displayed by these two closely related viruses [21],[50]. Future studies on the role of Tetherin antagonism in the pathogenesis of primate immunodeficiency virus will likely shed light on the contribution of this innate antiviral factor in the control of viral infection and spread in *vivo* and will reveal whether enhancing the antiviral activity of Tetherin is a valid option to thwart HIV-1 replication.

## Materials and Methods

### Ethics statement

Polyclonal rabbit antibodies against human Tetherin were produced according to animal experimentation research protocols approved by the Animal Care Committee of the Institut de recherches cliniques de Montreal and in accordance with Canadian Council on Animal Care (CCAC) guidelines and policies.

### Antibodies and chemical compounds

Anti-Vpu rabbit polyclonal serum was described previously [40]. Anti-Tetherin rabbit polyclonal serum was generated by immunization of rabbits with a bacterially-produced Glutathione-S-transferase (GST) fusion protein containing a polypeptide corresponding to amino-acids 40 to 180 of human Tetherin. Rabbit pre-immune serum was collected prior to rabbit immunization. Monoclonal anti-HA (clone 12CA5), anti-p24 (catalog no. HB9725) and anti-myc (clone 9E10) Abs were isolated from the supernatants of cultured hybridoma cells obtained from the American Type Culture Collection (ATCC). The monoclonal anti-gp120 antibody [51],[52] was obtained from NIH AIDS research and Reference Reagent Program. All secondary Alexa-conjugated IgG Abs were obtained from Invitrogen. Sheep anti-TGN46 (Serotec), mouse anti-BST2 (Abnova), anti-actin Abs (Sigma), pronase (Calbiochem), PNGase (New England Biolabs), BFA, paraformaldehyde (PFA) and chlorpromazine (Sigma) were all obtained from commercial sources. All reagents were stored according to the manufacturer's instructions.

### Cells and transfection

HEK 293T, HeLa and Cos-7 cells were obtained from ATCC. All cells were maintained as described previously [40]. HEK 293T and HeLa cells were transfected using the calcium-phosphate method and lipofectamine 2000 (Invitrogen), respectively. Functional and biochemical analyses were performed 48h post-transfection. Empty plasmid DNA was added to each transfection to keep the amount of transfected DNA constant.

### Plasmid constructs

HxBH10-*vpu+* and HxBH10-*vpu-* are two infectious molecular clones of HIV-1 that are isogenic except for the expression of Vpu [53]. HxBH10-*vpu S52D,S56D,* SVCMV*-vpu-* and SVCMV*-vpu+* were previously described, [25]. HxBH10-*vpu KSL*, HxB10-*vpu RD* and SVCMV-*vpu S52D,S56D* were generated by PCR-based site-directed mutagenesis [40]. The pcDNA/Myc-His-β-TrCP plasmid was kindly provided by Dr. Richard Benarous [27]. To generate pcDNA-Tetherin and pCMV-HA-Tetherin, the human Tetherin open reading frame was amplified by PCR from pCMV-SPORT6-hBST2 plasmid (Open Biosystems) and cloned into pcDNA3.1/Hygro (+) (Invitrogen) and pCMV-HA (Clontech). The agmTetherin open reading frame was amplified from Cos-7 cells mRNA using RT-PCR as described [29]. Tetherin chimeras were designed according to the structure prediction from Kupzig and Banting [4]. All Tetherin genes were inserted into pcDNA3.1/Hygro (+) using NheI and Asp718 restriction sites or into the pCMV-HA plasmid using BglII and Asp718 restriction sites to generate pcDNA-Tetherin and pCMV-HA-Tetherin constructs, respectively. pCMV-HA-Tetherin Y6Y8 and pCMV-HA-Tetherin ΔGI,T45I were generated by PCR-based site-directed mutagenesis. The vesicular stomatitis virus (VSV) glycoprotein G-expressing plasmid, pSVCMVin-VSV-G, was previously described [54]. The pQBI25 GFP-expressing plasmid was obtained from Qbiogene.

### Steady-state detection of proteins by western blot

Transfected HeLa or HEK 293T cells were lysed in CHAPS lysis buffer (50 mM Tris, 5 mM EDTA, 100 mM NaCl, 0.5% CHAPS, pH 7.2) or in radio-immunoprecipitation assay (RIPA-DOC) buffer (10 mM Tris pH 7.2, 140 mM NaCl, 8 mM Na_2_HPO_4_, 2 mM NaH_2_PO_4_, 1% Nonidet-P40, 0.5% sodium dodecyl sulfate, 1.2 mM deoxycholate), respectively. Proteins from lysates were resolved on 12.5% SDS-PAGE, electro-blotted and analyzed by western blot as described previously [40].

### Pulse-chase and radio-immunoprecipitation experiments

Pulse-chase experiments were perfomed as described previously [40]. Briefly, transfected HEK 293T or HeLa cells infected with VSV-G-pseudotyped HxBH10-derived virus at a MOI of 1 were pulse-labeled for 30 min and 2 h respectively, with 800 µCi/ml of [^35^S]methionine and [^35^S]cysteine (Perkin Elmer) and chased for different interval of times. Following lysis of radio-labeled cells in RIPA-DOC, lysates were first pre-cleared with protein A sepharose beads coated with pre-immune rabbit serum for 1 h. Pre-cleared cell lysates were then incubated with anti-Tetherin Abs for 2 h at 4°C prior to immunoprecipitation using protein A sepharose beads. In HEK 293T cells, Vpu proteins were sequentially immunoprecipitated using the same method. Labeled proteins were analyzed by SDS-PAGE and autoradiography.

### PNGase treatment

Cells were lysed in RIPA-DOC. Tetherin-containing lysates were incubated with anti-Tetherin Abs for 2 h at 4°C prior to addition of protein A sepharose beads. Following an incubation of 2 h at 4°C, beads were isolated by centrifugation, washed with denaturing buffer (New England Biolabs), resuspended in denaturing buffer and then boiled at 95°C for 10 min. The supplied reaction buffer was added along with NP-40 (0.1%) according to the manufacturer's suggestion. Samples were then digested with 1500 units of PNGase (New England Biolabs) at 37°C for 3 h. Control samples were incubated without the enzyme. Proteins were eluted from beads by boiling in an equal volume of sample buffer for 10 min and analyzed by immunoblotting.

### Virus release assay

The virus release assay was described previously [40]. Briefly, supernatants of transfected cells were clarified by centrifugation and filtered through a 45 µm filter. Viral particles were pelleted by ultracentrifugation onto a 20% sucrose cushion in PBS for 2 h at 130000 g at 4°C and lysed in RIPA-DOC. Gag products were analyzed by western blot. Viral release efficiency was evaluated by determining the ratio between the virion-associated Gag (p24) band signal and all intracellular Gag-related band signal using laser scanning densitometry.

### Production of VSVg-pseudotyped HIV-1 virus

HEK 293T cells were transfected with HxBH10 proviral constructs and pSVCMVin-VSV-G as described previously [54]. Supernatants of transfected cells were clarified, filtered and pelleted by ultracentrifugation as described above and resuspended in DMEM supplemented with 10% bovine serum (FBS). Viruses were titrated using a standard MAGI assay [54].

### Flow cytometry

Cells were washed in PBS, resuspended at a concentration of 1×10^6^cells/ml and stained with the specific anti-Tetherin serum for 45 min at 4°C. After incubation, cells were washed and stained using appropriate fluorochrome-coupled secondary Abs for 30 min at 4°C. Cells were then washed and fixed with 2% PFA. Transfected GFP-expressing cells were analyzed for cell-surface Tetherin expression by flow cytometry. Rabbit pre-immune serum served as a staining control. Fluorescence intensities were acquired using a FACScalibur flow cytometer (BD Biosciences) and data was analyzed using FlowJo software v. 7.25 (Treestar). MFI values presented in the histograms correspond to the specific signal obtained after substraction of the MFI value from the pre-immune control.

### Internalization assay

Cells were washed in PBS, re-suspended in PBS containing the anti-Tetherin serum at a concentration of 1×10^6^cells/ml and incubated for 45 minutes at 4°C. Following washes in cold PBS, cells were incubated at 37°C in DMEM medium supplemented with 5% FBS for different time intervals. At each time point, cells were harvested, washed in cold PBS and stained with the appropriate fluorochrome-coupled secondary Abs for 30 min at 4°C. Transfected GFP-expressing cells were analyzed for cell-surface Tetherin expression by flow cytometry.

### Cell-surface Tetherin re-expression assay

Cells were harvested, washed in PBS, re-suspended at a concentration of 1×10^6^ cells/ml in PBS-pronase 0.05% and incubated for 30 minutes at 37°C. Cold DMEM containing 10% FBS was added to block surface protein proteolysis. Cells were then washed, incubated at 37°C for different time intervals and stained for cell-surface Tetherin as described above. Expression of Tetherin at the cell-surface of transfected GFP-positive and untransfected GFP-negative cells was analyzed by flow cytometry.

### Confocal microscopy

HeLa cells were infected with VSV-G-pseudotyped HxBH10-derived viruses at a MOI of 0.125. Forty-eight hours post-infection, cells were immunostained with anti-Tetherin Abs (Abnova) for 30 min at 4°C, washed in cold PBS, fixed with 4% PFA and permeabilized with 0.2% Triton X-100. Next, cells were incubated with anti-Tetherin (Abnova), anti-Vpu and anti-TGN46 Abs for 2 h at 37°C, washed and incubated with the appropriate secondary Abs for 30 min at room temperature. Analyses were performed with a LSM710 laser scanning confocal microscope (Zeiss). Quantitation of Tetherin signal was performed using the Zeiss LSM510 software. The absolute Tetherin signal in the TGN was determined by measuring the specific Tetherin signal in the region delineated by the TGN46 marker on digital pictures produced using similar acquisition time. Percentage of Tetherin accumulating in the TGN was calculated by evaluating Tetherin signal intensity in the TGN relative to the total Tetherin signal intensity detected in the cell as described previously [40]. Analysis was performed on at least 25 distinct cells.

### Co-precipitation assay

For co-immnuoprecipitation of Vpu and Tetherin, transfected HEK 293T and HeLa cells were harvested 48 h post-transfection and lysed in RIPA-DOC or CHAPS buffer, respectively. Five percent of each lysates were preserved to control for protein expression. Cell lysates were first pre-cleared with protein A sepharose beads coated with pre-immune rabbit serum for 1 h at 4°C and then, incubated with anti-Tetherin Abs for 2 h at 4°C, prior to precipitation with protein A sepharose beads. Immunoprecipitates were analyzed for the presence of Vpu and Tetherin by western blot.

For co-immunoprecipitation of Vpu and β-TrCP, HEK 293T cells were transfected with SVCMV*-vpu-* or SVCMV*-vpu+ or* SVCMV-*vpu S52D,S56D and* pcDNA/Myc-His-β-TrCP. Transfected cells were then radiolabelled with 800 µCi/ml of [^35^S]methionine and [^35^S]cysteine (Perkin Elmer) and lysed in CHAPS buffer. Lysates were first pre-cleared with protein A sepharose beads coated with pre-immune rabbit serum for 1 h. Pre-cleared cell lysates were then incubated with anti-myc Abs for 2 h at 4°C prior precipitation using protein A sepharose beads. Vpu was then sequentially immunoprecipitated using anti-Vpu Abs using the same method. Labeled proteins were analyzed by SDS-PAGE and autoradiography.

### Scanning and quantitation

Scans were performed on a Duoscan T1200 scanner (AGFA) followed by densitometric quantitation using the Image Quant 5.0 software (Molecular Dynamics). Statistical analysis was performed using a paired Student's *t* test, and statistical significance was considered at p<0.001.

### Accession numbers

NCBI reference number for HxBH10 Vpu and human Tetherin proteins are P69699 and AAH33873, respectively. Genebank accession number for agm Tetherin is FJ943430.

## Supporting Information

Figure S1 Characterization of the Vpu S52D,S56D mutant. (A) Association of Vpu with β-TrCP. HEK 293T cells were transfected with the indicated Vpu-expressing constructs and the myc-β-TrCP-encoding plasmid pcDNA/Myc-His-β-TrCP. Forty-eight hours post-transfection, cells were radio-labeled for 2h and lysed prior to sequential immunoprecipitation using anti-myc and, subsequently, anti-Vpu Abs. Co-immunoprecipitated proteins were separated by SDS-PAGE and analyzed by autoradiography. (B) Effect of Vpu S52D,S56D on Tetherin cell-surface expression. HeLa cells were transfected with the indicated HxBH10 proviral constructs and a GFP-expressing plasmid. Cell-surface Tetherin expression was analyzed on GFP-positive cells by flow cytometry, 48h post-transfection. MFI values are shown beside the histogram. Filled histogram: pre-immune control; dashed line: HxBH10-*vpu*-; full black line: HxBH10-*vpu*+; full grey line: HxBH10-*vpu S52D,S56D*. (C) Effect of Vpu S52D,S56D on HIV-1 particle release. HeLa cells were transfected with the indicated HxBH10 proviral constructs. Cells and virus-containing supernatants were collected 48h post-transfection, lysed and analyzed for the detection of Gag-related products and Vpu by western blot using specific Abs. (D) Quantitation of virus particle release. Bands corresponding to Gag products in cells and virus particles were scanned by laser densitometry. The relative virus particle release efficiency was determined as described in the Materials and Methods and calculated as a percentage of the HxBH10-*vpu*+ virus release (100%). The error bars represent the standard deviation from the mean of three independent experiments.
(0.35 MB TIF)

Figure S2 Functional analysis of Tetherin chimeric proteins and the ΔGI,T45I mutant. (A) Design of Tetherin chimeric proteins and the human Tetherin ΔGI-T45I mutant. Cytoplasmic (Cyto), TM, coiled-coil and GPI domain as well as glycosylation and cleavage sites (dashed line) are represented. White: human Tetherin; grey: agm Tetherin. The amino-acid sequence of the mutant Tetherin ΔGI,T45I within the TM domain is shown below. Dots indicate deleted residues while hyphens indicate similar residues. (B-E) HEK 293T cells were transfected with the specified HxBH10 proviral constructs and the indicated plasmids expressing (B) native or (D) HA-tagged Tetherin proteins. Forty-eight hours post-transfection, cells and virus-containing supernatants were harvested, lysed and proteins were analyzed by western blot using specific Abs. (C and E) Quantitation of B and D, respectively. Bands corresponding to Gag products in cells and viral particles of panels B or D were scanned by laser densitometry. The virus particle release efficiency was determined as described in the Materials and Methods and calculated as a percentage of the HxBH10-*vpu*+ release (100%) in absence of ectopically-expressed Tetherin. Error bars represent the standard deviation from the mean of two independent experiments.
(0.82 MB TIF)

Figure S3 Functional analysis of Vpu mutants in HEK 293T cells. HEK 293T cells were transfected with plasmids encoding native Tetherin, the indicated HxBH10 proviral constructs and a GFP-expressing plasmid. (A) Forty-eight hours post-transfection, cells and virus-containing supernatant were harvested, lysed and proteins were analyzed by western blot using specific Abs. (B) In parallel, cell-surface Tetherin expression was analyzed on GFP-positive cells by flow cytometry. Geo mean values (depicted as MFI) are presented in the histograms. Filled histogram: pre-immune control; dashed line: HxBH10-*vpu*-; full black line: HxBH10-*vpu*+; dotted line: HxBH10-*vpu KSL*; full grey line: HxBH10-*vpu RD*.
(0.42 MB TIF)
