# Supplementary figures and images for: Antagonism of Tetherin Restriction of HIV-1 Release by Vpu Involves Binding and Sequestration of the Restriction Factor in a Perinuclear Compartment
Source: PLoS Pathog. 2010 Apr 8;6(4):e1000856. doi: 10.1371/journal.ppat.1000856 (PMC2851737; doi:10.1371/journal.ppat.1000856)

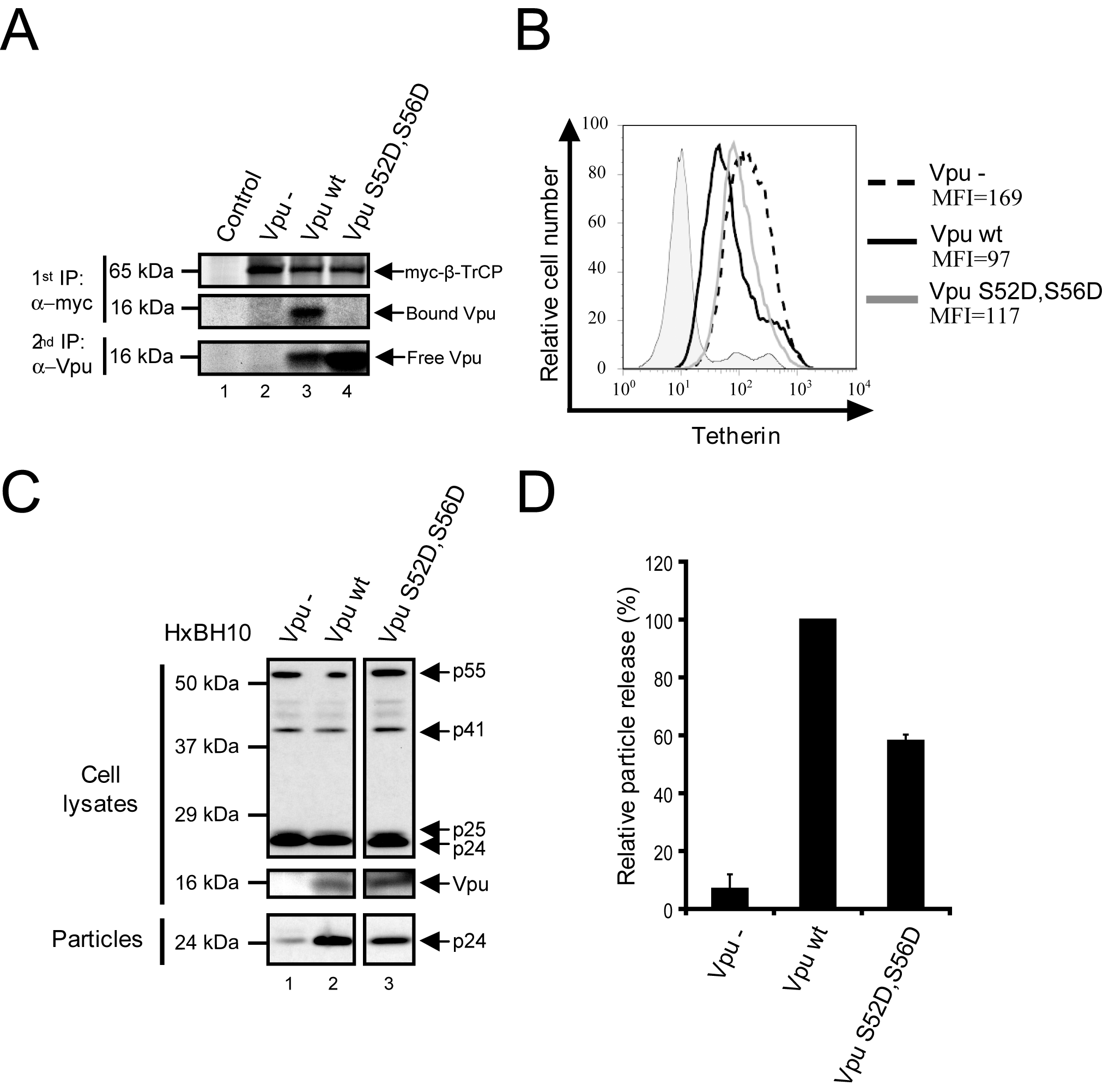

Supplement: Figure S1 — Characterization of the Vpu S52D,S56D mutant. (A) Association of Vpu with β-TrCP. HEK 293T cells were transfected with the indicated Vpu-expressing constructs and the myc-β-TrCP-encoding plasmid pcDNA/Myc-His-β-TrCP. Forty-eight hours post-transfection, cells were radio-labeled for 2h and lysed prior to sequential immunoprecipitation using anti-myc and, subsequently, anti-Vpu Abs. Co-immunoprecipitated proteins were separated by SDS-PAGE and analyzed by autoradiography. (B) Effect of Vpu S52D,S56D on Tetherin cell-surface expression. HeLa cells were transfected with the indicated HxBH10 proviral constructs and a GFP-expressing plasmid. Cell-surface Tetherin expression was analyzed on GFP-positive cells by flow cytometry, 48h post-transfection. MFI values are shown beside the histogram. Filled histogram: pre-immune control; dashed line: HxBH10-vpu-; full black line: HxBH10-vpu+; full grey line: HxBH10-vpu S52D,S56D. (C) Effect of Vpu S52D,S56D on HIV-1 particle release. HeLa cells were transfected with the indicated HxBH10 proviral constructs. Cells and virus-containing supernatants were collected 48h post-transfection, lysed and analyzed for the detection of Gag-related products and Vpu by western blot using specific Abs. (D) Quantitation of virus particle release. Bands corresponding to Gag products in cells and virus particles were scanned by laser densitometry. The relative virus particle release efficiency was determined as described in the Materials and Methods and calculated as a percentage of the HxBH10-vpu+ virus release (100%). The error bars represent the standard deviation from the mean of three independent experiments. (0.35 MB TIF) [file ppat.1000856.s001.tif]

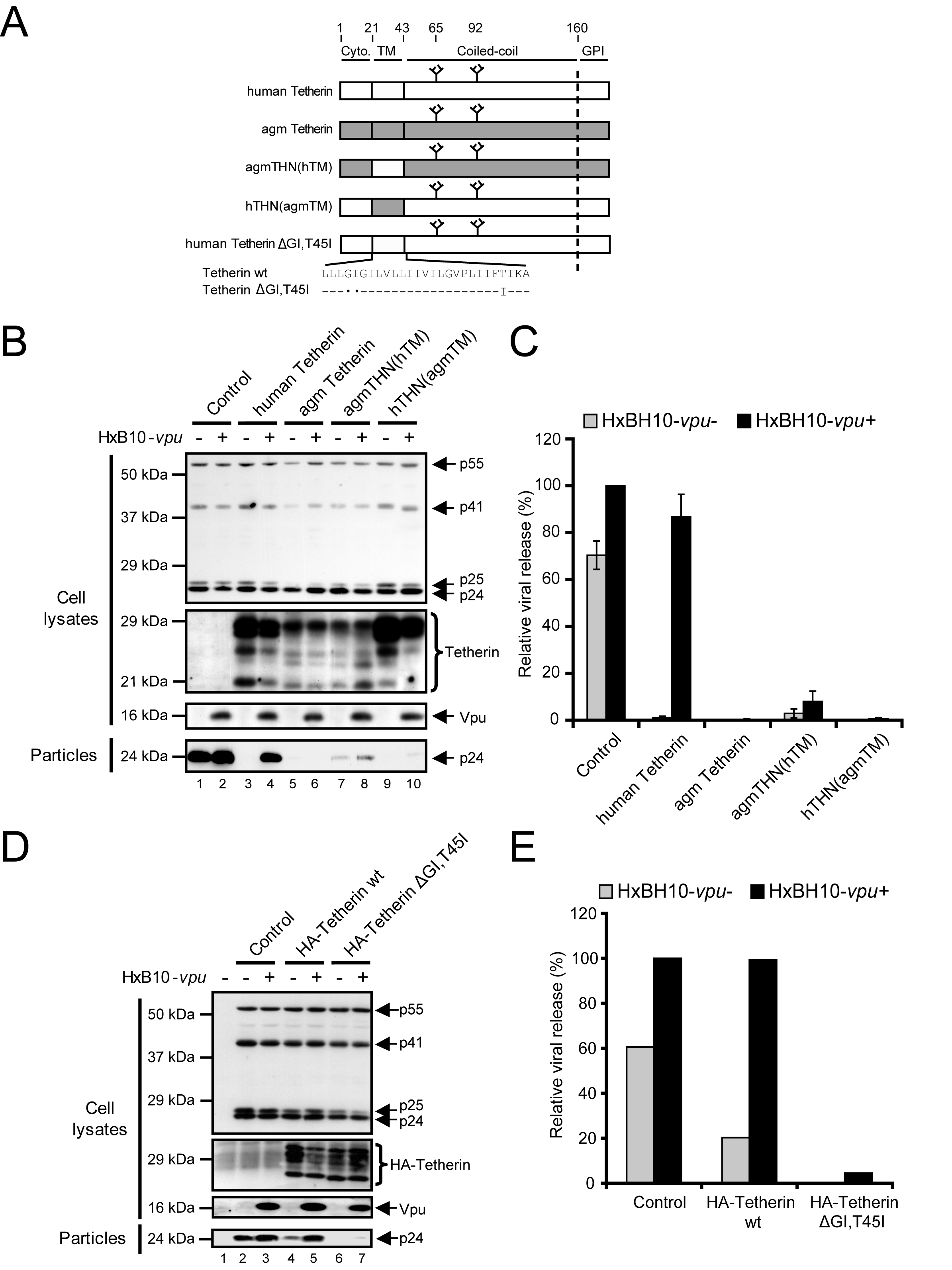

Supplement: Figure S2 — Functional analysis of Tetherin chimeric proteins and the ΔGI,T45I mutant. (A) Design of Tetherin chimeric proteins and the human Tetherin ΔGI-T45I mutant. Cytoplasmic (Cyto), TM, coiled-coil and GPI domain as well as glycosylation and cleavage sites (dashed line) are represented. White: human Tetherin; grey: agm Tetherin. The amino-acid sequence of the mutant Tetherin ΔGI,T45I within the TM domain is shown below. Dots indicate deleted residues while hyphens indicate similar residues. (B-E) HEK 293T cells were transfected with the specified HxBH10 proviral constructs and the indicated plasmids expressing (B) native or (D) HA-tagged Tetherin proteins. Forty-eight hours post-transfection, cells and virus-containing supernatants were harvested, lysed and proteins were analyzed by western blot using specific Abs. (C and E) Quantitation of B and D, respectively. Bands corresponding to Gag products in cells and viral particles of panels B or D were scanned by laser densitometry. The virus particle release efficiency was determined as described in the Materials and Methods and calculated as a percentage of the HxBH10-vpu+ release (100%) in absence of ectopically-expressed Tetherin. Error bars represent the standard deviation from the mean of two independent experiments. (0.82 MB TIF) [file ppat.1000856.s002.tif]

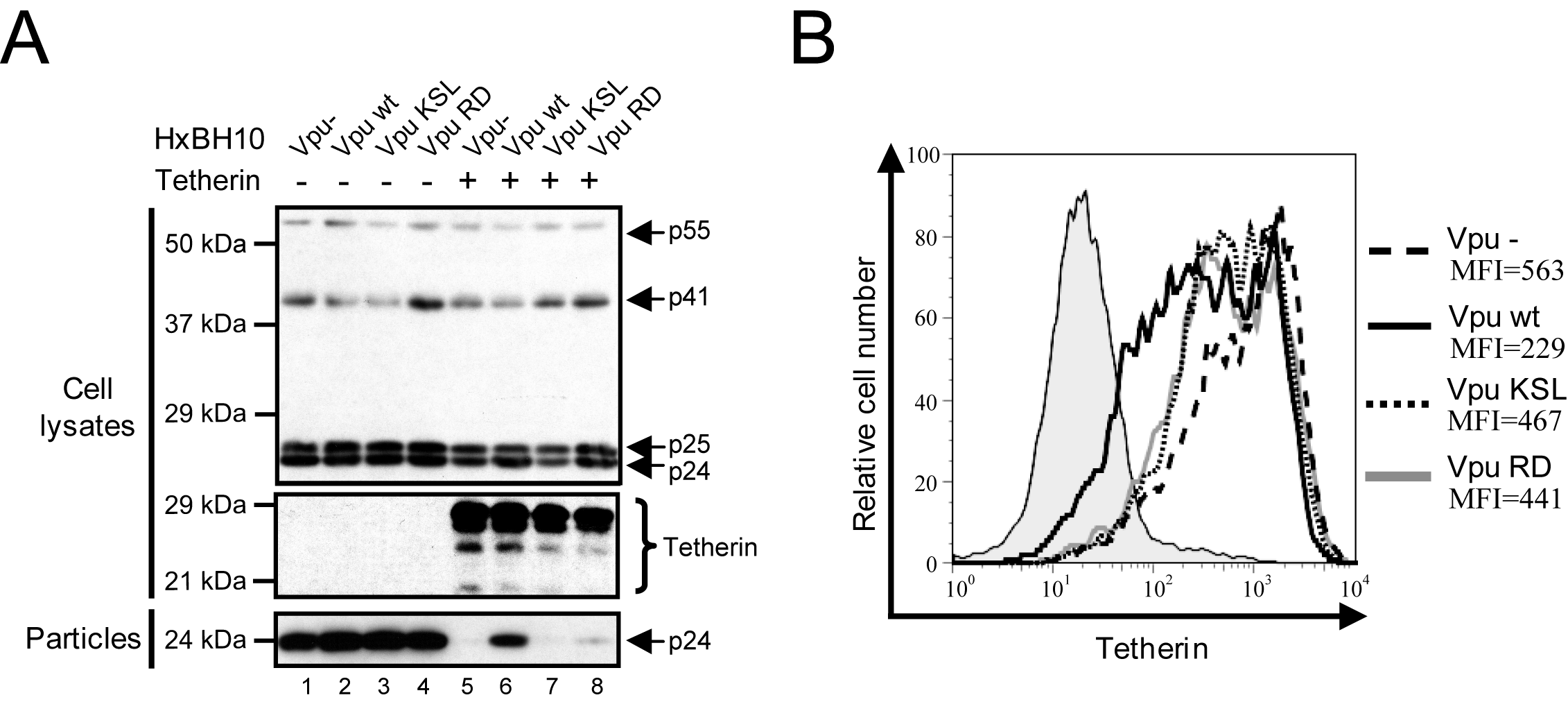

Supplement: Figure S3 — Functional analysis of Vpu mutants in HEK 293T cells. HEK 293T cells were transfected with plasmids encoding native Tetherin, the indicated HxBH10 proviral constructs and a GFP-expressing plasmid. (A) Forty-eight hours post-transfection, cells and virus-containing supernatant were harvested, lysed and proteins were analyzed by western blot using specific Abs. (B) In parallel, cell-surface Tetherin expression was analyzed on GFP-positive cells by flow cytometry. Geo mean values (depicted as MFI) are presented in the histograms. Filled histogram: pre-immune control; dashed line: HxBH10-vpu-; full black line: HxBH10-vpu+; dotted line: HxBH10-vpu KSL; full grey line: HxBH10-vpu RD. (0.42 MB TIF) [file ppat.1000856.s003.tif]
